# An EBNA1-YAP signaling axis drives immune escape through CD276 in EBV-associated gastric cancer

**DOI:** 10.1038/s41419-025-08251-2

**Published:** 2025-12-19

**Authors:** Binhao Huang, Mengqi Liu, Yantao Duan, Jing Guo, Zixian Wang, Yi Dou, Mengyun Wang, Omar Abuhaidar, Henian Sun, Leung Siu Kee, Yu Wang, Gong-Hong Wei, Dazhi Xu

**Affiliations:** 1 Department of Gastric Surgery, Fudan University Shanghai Cancer Center, Fudan University, Shanghai, China; 2 Department of Oncology, Shanghai Medical College, Fudan University, Shanghai, China; 3 MOE Key Laboratory of Metabolism and Molecular Medicine and Department of Biochemistry and Molecular Biology of School of Basic Medical Sciences, and Fudan University Shanghai Cancer Center, Shanghai Medical College of Fudan University, Shanghai, China; 4 State Key Laboratory of Common Mechanism Research for Major Diseases, Suzhou Institute of Systems Medicine, Chinese Academy of Medical Sciences & Peking Union Medical College, Suzhou, Jiangsu China; 5 N.N. Blokhin National Medical Research Center of Oncology of Russia, Moscow, Russia; 6 Department of Surgery, Tuen Mun Hospital, Hong Kong, China

**Keywords:** Tumour biomarkers, Predictive markers, Gastric cancer

## Abstract

Clinical efficacy of anti-PD1 immunotherapy often yields low response rates in Epstein-Barr virus-associated gastric cancer (EBVaGC). To gain insights into immune escape mechanisms and discover critical molecules in anti-tumor immunity, we performed an immune checkpoint screening using transcriptome profiling and immunohistochemistry analyses. We identified CD276 as an independent immunosuppressive molecule that correlates with poor EBVaGC prognosis. Our in vitro and in vivo experiments demonstrate the role of CD276 in inducing T cell apoptosis and diminishing chemokine secretion, thereby dampening immune response and facilitating tumor progression. Mechanistically, we discovered that YAP/TEAD4 chromatin occupancy at CD276 regulatory regions leads to its transcriptional upregulation in EBVaGC, driven by EBNA1-stimulated MST1/2-LATS1/2-YAP signaling. Notably, in a humanized xenograft mouse model, EBVaGC with elevated CD276 levels exhibited resistance to anti-PD1 immunotherapy, while targeting CD276 in combination with PD1 blockade significantly reduced tumor size. Collectively, our findings elucidate the EBNA1-YAP-CD276 axis as a novel mechanism of immune escape in EBVaGC, providing insights for enhanced immunotherapeutic strategies.

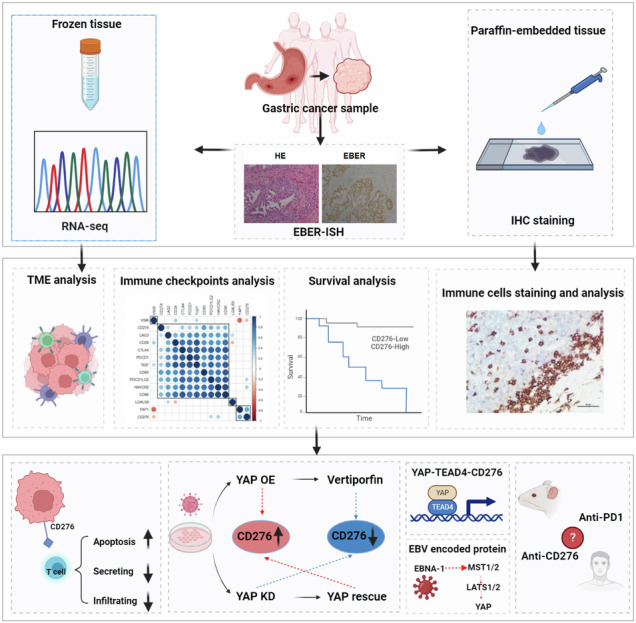

## Introduction

Gastric cancer (GC) is among the most prevalent malignancies of the digestive system and ranks as the third leading cause of cancer-related mortality worldwide. Despite the widespread use of chemotherapy, patients with advanced GC experience an overall survival of only about one year [1]. The advent of cancer immunotherapy, particularly with PD-1/PD-L1 inhibitors, has transformed treatment options for various solid tumors [2]. However, their effectiveness in GC remains disappointingly low, with response rates around 10% [3–6]. This highlights an urgent need for innovative therapeutic targets to improve the efficacy of immunotherapy for GC patients.

Epstein-Barr virus-associated gastric cancer (EBVaGC) represents a distinct subtype of GC, characterized by a unique tumor microenvironment shaped by viral infection and chronic inflammation. This environment is marked by significant immune cell infiltration, particularly CD8+ T cells, which are known for their anti-tumor activity [7–9]. Given these features, EBVaGC is anticipated to respond well to immunotherapy. However, response rates to anti-PD-1 therapies in EBVaGC patients are highly variable [10, 11], with partial response rates rarely exceeding 30% [3, 12–14]. Our prior research into the immune landscape of EBVaGC revealed that only approximately 53% (26/50) of cases exhibit signs of immune activation, which may be partially linked to ASTE1 mutations [15]. Alarmingly, nearly half of the EBVaGC cases in our cohort display immune inactivity, with the molecular mechanisms behind this phenomenon remaining unclear [15]. Additional studies support these observations, showing that a significant portion of EBVaGC cases have low lymphocyte infiltration and lack PD-L1 expression [16, 17]. The development of an immunosuppressive tumor microenvironment (TME) and the emergence of “cold tumors” further complicate the efficacy of immunotherapy in EBVaGC. To address these challenges, it is crucial to elucidate the underlying mechanisms of immune escape in EBVaGC. This knowledge will be instrumental in optimizing immunotherapeutic strategies and ultimately improving outcomes for patients afflicted by this aggressive disease.

CD276 (B7-H3) is a relatively novel immune checkpoint molecule within the B7 family, recognized as an orphan ligand that plays dual roles in immune modulation, exhibiting both co-stimulatory and co-inhibitory effects [18–20]. Emerging evidence highlights its significant role as an immunosuppressive factor in various malignancies [21]. CD276 is frequently overexpressed in many human cancers and correlates with poor prognostic outcomes [22, 23]. Inhibiting CD276 has shown promise in enhancing T cell activation and significantly increasing CD8+ T cell infiltration [24, 25]. Despite these findings, the specific immunoregulatory functions and clinical relevance of CD276 in EBVaGC remain largely unexamined. Investigating this molecule could unveil critical insights into its role in immune evasion and therapeutic potential in EBVaGC.

Aberrant expression of critical cancer driver genes, often regulated by dysregulated upstream signaling pathways, is a hallmark of cancer development [26]. In EBVaGC, EBV infection triggers the nuclear translocation of Yes-associated protein (YAP), a key transcriptional co-activator and effector of the Hippo signaling pathway, which plays a vital role in tumor suppression, tissue homeostasis, and organ size regulation [27–29]. Emerging evidence implicates YAP in modulating immune responses and facilitating immune evasion. For instance, components of the Hippo pathway, including TAZ, YAP, and TEAD, have been shown to enhance the expression of immune-suppressive molecules by directly binding to their promoters, promoting immune escape in cancers such as melanoma and breast cancer [30, 31]. This raises the important question of whether similar mechanisms underlie CD276 overexpression and consequent immune escape in EBVaGC. Investigating these pathways could provide valuable insights into the immunological landscape of this cancer subtype and inform potential therapeutic strategies.

In this study, we performed a comprehensive analysis of whole transcriptome sequencing (RNA-seq) and immune profiling data from a cohort of 50 clinically and pathologically characterized EBVaGC patients. Our findings highlight CD276 as a critical and independent immune checkpoint molecule, significantly modulating various immune responses in both physiological and pathological contexts, and correlating with poor prognostic outcomes in EBVaGC. Furthermore, we identified that the overexpression of CD276 in EBVaGC is primarily regulated by YAP activation, which is triggered by the EBV-encoded EBNA1, thereby revealing a novel upstream regulatory mechanism specific to this cancer subtype.

## Results

### Immune infiltration suppressive function of CD276 with prognostic potential in EBVaGC

**Fig. 1:**
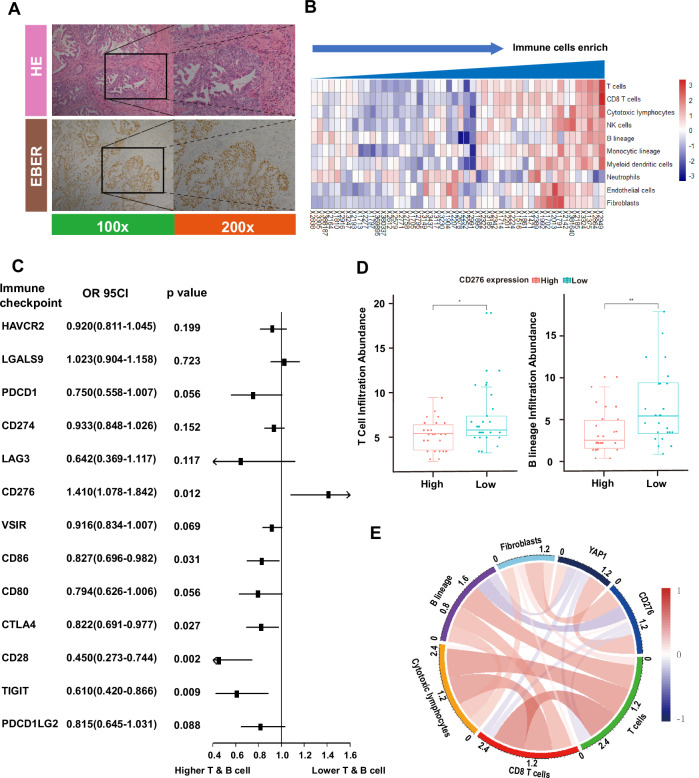
Prognostic value and immunosuppressive function of CD276 revealed by transcriptome sequencing of EBVaGC. **A** Histopathological determination of EBVaGC. Hematoxylin andeosin (H&E) staining (upper) and the EBV-encoded RNA (EBER) in situ hybridization (lower) at the same location of two continuous slices; magnification 100x (left) and 200x (right). **B** Immune cells infiltration score in tumor microenvironment was predicted by the “MCP-Counter” R package from EBVaGC RNA-sequencing data. The enrichment of different immune cells in 50 EBVaGC was listed. **C** Logistic regression model for risk factors towards lower tumor-infiltrating lymphocyte levels. Note that CD276 was the only immune checkpoint molecule inducing fewer infiltrating T and B cells (*p* = 0.012, OR = 1.410). **D** The CD276-high group had fewer infiltrating T cells and B lineages (*p* < 0.05 and 0 < 0.01). **E** Circos plot for the correlation between CD276 and other immune cell components derived from the MCP-counter approach.

We performed transcriptome profiling of 50 EBVaGC tumor specimens paired with adjacent normal tissues via RNA sequencing (RNA-seq). Of the 50 patients, 46 were male and 4 were female (Supplementary Table 1). To confirm EBV involvement, typical in situ hybridization for EBV-encoded RNA (EBER-ISH) was performed, complemented by Hematoxylin and Eosin (H&E) staining as a negative control, demonstrating strong nuclear staining in cancer cells (Fig. 1A). Thus, all patient tumors in this study were confirmed to be EBER-ISH–positive.

To assess immune regulation within the EBVaGC tumor microenvironment (TME), we first evaluated the cellular composition using MCP-counter [32], which provided absolute abundance scores for various tumor-infiltrating immune cells (Fig. 1B). Subsequently, we conducted a comprehensive analysis of several common immune checkpoint molecules [33] (including HAVCR2, LGALS9, PDCD1, CD274, LAG3, VSIR, CD86, CD80, CTLA4, CD28, TIGIT, PDCD1LG2, and CD276) to further investigate immune modulation. Higher expression of most immune checkpoints was associated with increased T and B lymphocyte infiltration, as indicated by logistic regression analysis (Fig. 1C). Notably, CD276 emerged as a distinct immune checkpoint molecule with significant effects on immune infiltration, functioning as a unique risk factor for lymphocyte infiltration (*p* = 0.012, OR = 1.410) (Fig. 1C). Specifically, EBVaGC tumors with high CD276 expression exhibited reduced infiltration of T and B lineage cells (Fig. 1D), fewer CD8 T cells, and a comparable presence of cytotoxic lymphocytes, NK cells, monocytic lineages, myeloid dendritic cells, neutrophils, and endothelial cells. In contrast, fibroblasts infiltration was higher in CD276-High tumors (Fig. 1E and Supplementary Fig. 1A).

**Fig. 2:**
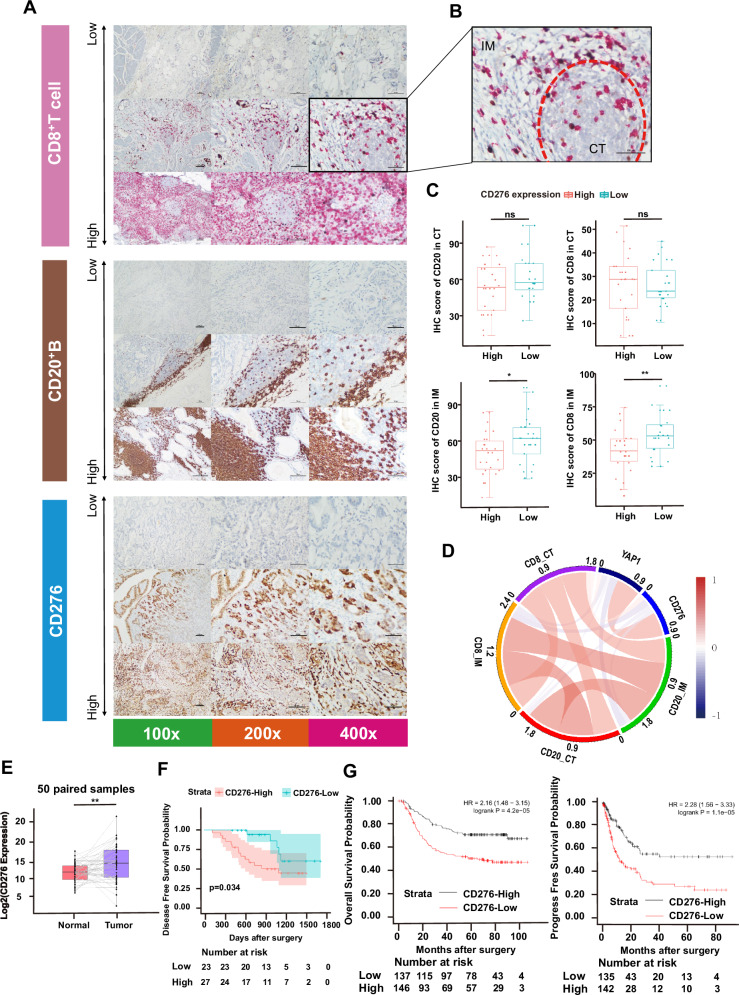
CD276 expression correlates with CD8 + T cell and CD20 + B cell infiltration in EBVaGC. **A** Representative images of immunohistochemistry (IHC) staining presented by 100x, 200x and 400x (left, middle and right, respectively). Scale bar, 100 µm. **B** The staining score was calculated in the center of the tumor (CT) and the invasive margin (IM), respectively. **C** The CD276-high group showed significantly lower IHC scores as the surrogates for CD8+ T cell or CD20+ B cell only in IM but not in CT. **D** Circos plot for the correlation between CD276 and other immune cell components derived from IHC. **E** Comparison of CD276 expression between EBVaGC tumors and paired normal tissues. Tumors expressed a higher level of CD276 (*p* < 0.01). **F** Prognostic value of CD276 in EBVaGC. The CD276-High group had a significantly worse DFS than the CD276-Low group (*n* = 50, *p* = 0.034). **G** Validation of the prognostic value of CD276 in GSE62254 (GEO database). The CD276-High group had worse OS (upper, *n* = 283, *p* < 0.001) and DFS (lower, *n* = 275, *p* < 0.001).

CD276, a relatively novel immune checkpoint molecule, plays a pivotal role in modulating tumor-immune interactions [21]. To further investigate the relationship between CD276 and the immune microenvironment, we performed immunohistochemistry (IHC) staining for representative immune cell types (Fig. 2A) and analyzed the IHC scores at both the tumor center (CT) and invasive margin (IM) (Fig. 2B). We observed that the CD276-High group displayed significantly lower IHC scores for CD3- and CD8-positive T cells at the IM, but not at the CT (Fig. 2C, D). No significant differences were noted for other immune cell types, such as M1 and M2 macrophages or Tregs (Supplementary Fig. 1B). These findings highlight the spatial heterogeneity of CD276’s immune-suppressive function, particularly at the tumor invasive margin [34] (Fig. 2C).

Next, differential gene expression analysis revealed 173 upregulated and 168 downregulated genes in the CD276-High group compared to the CD276-Low group (*p* < 0.05, |logFC| > 1) (Supplementary Fig. 2A). The Kyoto Encyclopedia of Genes and Genomes (KEGG) pathway analysis identified the “PI3K-Akt signaling pathway”, “Human papillomavirus infection”, and “Hippo signaling pathway” as significantly enriched in the CD276-High group (Supplementary Fig. 2B). A more expansive analysis of 1792 upregulated genes revealed enriched immune-related pathways, including “cytokine–cytokine receptor interaction”, “viral protein interaction with cytokine and cytokine receptor”, and “IL-17 signaling pathway” (Supplementary Fig. 2C). Notably, several chemokines (CCL7, CXCL1, CXCL5, CXCL8, IL1, IL11, IL24) were upregulated in the CD276-High tumors (Supplementary Fig. 2A).

We also compared CD276 expression between EBVaGC tumor specimens and adjacent normal tissues. Tumors exhibited significantly higher expression of CD276 than paired normal tissue (*p* = 0.006) (Fig. 2E), suggesting an upregulation of CD276 during gastric tumorigenesis.

To explore the prognostic value of CD276, we conducted survival analysis using comprehensive follow-up and clinical outcome data from our cohort. The optimal cutoff for CD276 expression was determined by selecting the maximal standardized log-rank statistic (Supplementary Fig. 3). Using a cutoff of FPKM as 6, 27 patients in the CD276-High group demonstrated significantly worse disease-free survival (DFS) (*p* = 0.034) (Fig. 2F). After adjusting for TNM stage in a multivariate Cox regression model, CD276 expression remained significantly associated with poor prognosis (*p* = 0.034, HR = 3.023). A comparison of baseline characteristics revealed larger tumor sizes in the CD276-High group, while no significant differences in sex, age, tumor location, or Lauren classification (Supplementary Table 1).

Further analysis of clinical data from a large independent GC cohort (GSE62254, GEO database) [35] corroborated our findings, showing worse overall survival (OS) and DFS in the CD276-High group (*p* < 0.001) (Fig. 2G), reinforcing the prognostic significance of CD276 in EBVaGC.

### Immunosuppressive function of CD276 in vitro and in vivo

**Fig. 3:**
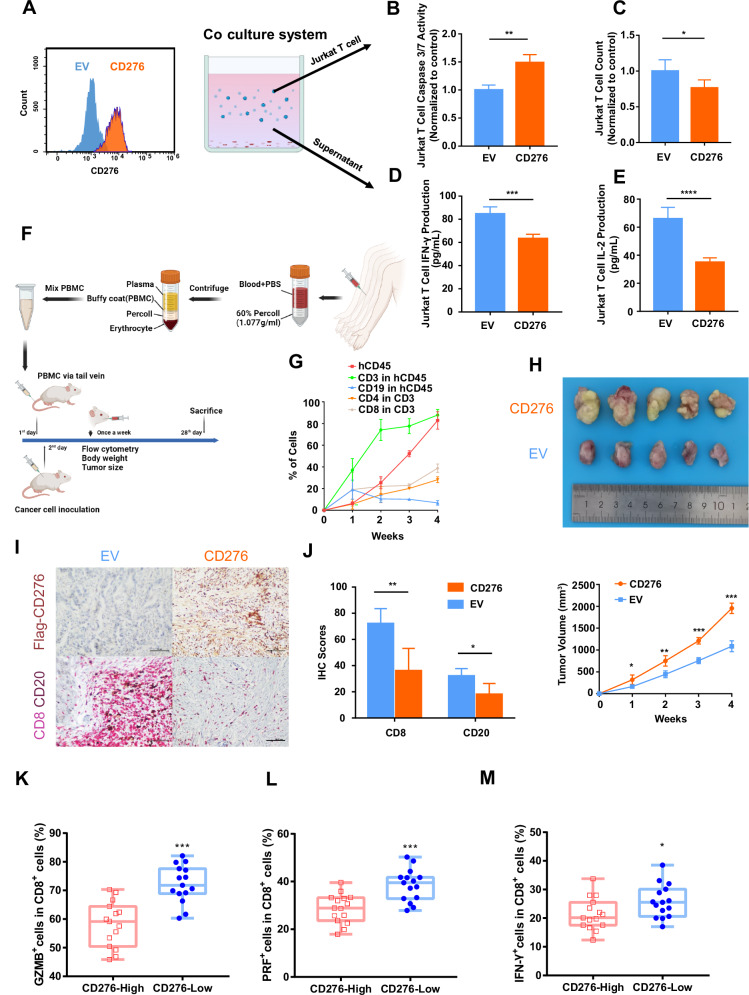
Immunosuppressive function of CD276 in vitro and in vivo. **A** Cytometry validation of CD276 overexpression in SNU-719 cell line. The cancer cells were co-cultured with activated Jurkat T cells for 48 h in vitro, and the T cells and co-culture supernatants were collected for apoptosis and secreting assay. EV: empty vector. **B** Apoptosis detection of Jurkat T cells after co-culture. Caspase 3/7 activity of T cells cultured with CD276 overexpression cancer cells was normalized to that of T cells cultured with empty vector transfected cancer cells. **C** T cells counting after co-culture with cancer cells. **D** IFN-γ secretion assay in co-culture supernatants. **E** IL-2 secretion assay in co-culture supernatants. **F** The schematic flow chart of xenograft model with immune system humanized mouse. **G** The proportion of each immune cell in the peripheral blood of humanized mice was detected every week by flow cytometry. **H** The tumor masses moved from xenograft model (left). The tumor volume of the mice was measured every week (right). Tumors grew faster in CD276 overexpressed group. **I** The infiltration of CD8+ and CD20+ lymphocytes in mouse tumor tissues was examined (200x). **J** CD276-overexpressed group had a lower lymphocyte IHC scores. **K–M** Flow cytometry using fresh tissue from 15 EBVaGC indicated that infiltrating CD8 T cells in CD276-High group expressed lower GZMB, PRF, and IFN-γ.

We next investigated the immunosuppressive function of CD276 in the EBVaGC tumor microenvironment, both in vitro and in vivo. In our in vitro experiments, SNU-719 cells (EBV + ) were engineered to overexpress CD276 and co-cultured with activated Jurkat T cells (Fig. 3A). The results showed that CD276 overexpression led to increased apoptosis in T cells and a concurrent reduction in their overall numbers (Fig. 3B, C). Moreover, we observed decreased levels of IFN-γ and IL-2 in the supernatant of the co-culture system, suggesting that CD276 may not only promote apoptosis but also inhibit T cell cytokine secretion (Fig. 3D, E).

To further explore the role of CD276 in the immune microenvironment in vivo, we employed a humanized mouse model. Specifically, we infused human peripheral blood mononuclear cells (PBMCs) via tail vein into NOD-SCID IL2Rγ^null^ mice to reconstruct the human immune system (Fig. 3F). The following day, we subcutaneously inoculated the mice with stable EBVaGC SNU-719 cells transfected with either CD276 or an empty vector to establish xenograft models. To assess the immune system reconstruction, we monitored the peripheral blood of the mice (Fig. 3G and Supplementary Fig. 4A) and measured their body weight weekly (Supplementary Fig. 4B). Throughout the study period, the levels of human immune cells in the mouse blood remained high, and no significant signs of xenogenic graft-versus-host disease (xeno-GvHD) were observed.

Our in vivo results demonstrated that CD276 overexpression accelerated tumor growth, with notably larger tumors observed at the 4-week time point compared to the control group (Fig. 3H). Additionally, CD276 overexpression was associated with a reduction in the infiltration of CD8^+^ and CD20^+^ immune cells into the tumor (Fig. 3I, J), further suggesting that CD276 exerts an immunosuppressive effect in the TME.

Finally, to corroborate our findings in clinical samples, we prospectively collected fresh tissue samples from 15 EBVaGC patients and analyzed immune cell profiles using flow cytometry. These samples were stratified based on CD276 expression levels. In the CD276-High group, infiltrating CD8 T cells exhibited significantly reduced expression of granzyme B (GZMB), perforin (PRF), and IFN-γ (Fig. 3K–M and Supplementary Fig. 4C), indicating a suppressed anti-tumor immune response in these patients.

### YAP-TEAD4 contributes to transcriptional upregulation of CD276 in EBVaGC

**Fig. 4:**
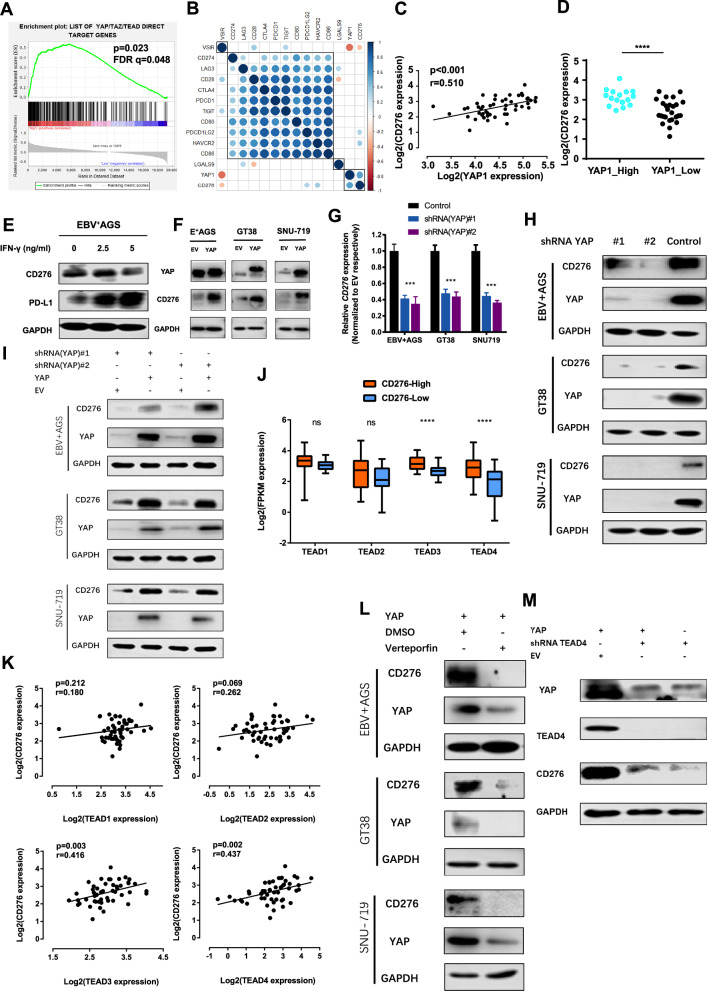
Independent regulation mode of CD276 and its association with YAP/TEAD4 signaling. **A** Gene set enrichment analysis (GSEA) showing significant enrichment of YAP/TAZ/TEAD direct target genes in the CD276-High group. **B** Expression correlation clustering of immune checkpoint molecules and YAP1, with significant negative correlations marked in blue and positive correlations in red. Larger dot size indicates smaller *p* values. **C** CD276 expression positively correlated with that of YAP1. **D** The YAP1-High group showed higher expression levels of CD276 (*p* < 0.001). **E** IFN-γ stimulation test in vitro. PD-L1 expression was upregulated as the stimulation dose of IFN-γ increased while CD276 expression could not be effectively induced by IFN-γ. **F** Overexpression of YAP in three EBV-positive cell lines increased CD276 expression. **G** qPCR revealed that CD276 mRNA expression was decreased when YAP was knocked down by shRNA#1 and #2 in three cell lines. **H** Immunoblot analysis confirming decreased expression of YAP and CD276 upon YAP knockdown. **I** Restoration of CD276 expression in YAP-knockdown cell lines by reintroducing YAP. **J** CD276-High group exhibited higher TEAD3 and TEAD4 expression (*p* < 0.001) but similar levels of TEAD1 and TEAD2 compared to the CD276-Low group. **K** Positive correlation of TEAD3 (p = 0.003, *r* = 0.416) and TEAD4 (*p* = 0.002, *r* = 0.437) expression with CD276, whereas TEAD1 and TEAD2 showed no significant correlation. **L** Verteporfin treatment, which inhibits YAP-TEAD interactions, decreased the expression of both YAP and CD276. **M** Knocking down TEAD4 reduced the expression of YAP and CD276.

Our analysis of sequencing data, along with a previously published gene set by Jiao and colleagues [28], revealed significant enrichment of “YAP/TAZ/TEAD direct target genes” in the CD276-High group of EBVaGC tumors (Fig. 4A). Subsequent correlation analysis demonstrated a positive relationship between YAP and CD276 expression, independent of other immune checkpoint molecules (Fig. 4B). These findings suggest that the YAP-TEAD complex may play a crucial role in the transcriptional regulation of CD276.

We next compared the involvement of YAP and TAZ, the paralogs of the YAP-TEAD pathway, in regulating CD276 expression. Our analysis indicated that YAP1, rather than TAZ, co-expressed with CD276 (Fig. 4C and Supplementary Fig. 5A). Notably, tumors with higher CD276 expression also exhibited significantly higher levels of YAP expression, whereas TAZ expression showed no significant difference between the groups (Supplementary Fig. 5B). Further validation using the SNU-719 cell line showed that YAP overexpression led to increased CD276 RNA expression, while TAZ overexpression did not significantly affect CD276 levels (Supplementary Fig. 5C).

Our clinical data revealed that upregulation of YAP is associated with poorer prognosis in EBVaGC patients (Supplementary Fig. 5). As YAP functions centrally within the Hippo signaling pathway (Supplementary Fig. 6A–C), we explored whether the expression of CD276 and YAP was correlated. Indeed, our data indicated that CD276 expression was positively correlated with YAP1 expression (Figs. 1E and 4B, C, *p* < 0.001, *r* = 0.510), suggesting concurrent high expression of both genes in EBVaGC tumors. Additionally, CD276 expression could predict YAP1 level with high accuracy, as determined by receiver operating characteristic (ROC) analysis (AUC = 0.858, sensitivity = 64.7%, specificity = 93.6%) (Supplementary Fig. 6D). Furthermore, tumors with high YAP1 expression also exhibited elevated CD276 levels (*p* < 0.001) (Fig. 4D). Similar results were obtained from the TCGA gastric cancer datasets (Supplementary Fig. 7), supporting the regulatory relationship between YAP1 and CD276 in EBVaGC.

Considering the role of IFN-γ as a potent inducer of various immunosuppressive molecules, we explored its effect on CD276 expression in EBV-positive GC cell lines. While IFN-γ treatment resulted in the upregulation of PD-L1 expression, no significant induction of CD276 expression was observed (Fig. 4E). These findings suggest that CD276 is regulated by a distinct mechanism, independent of IFN-γ signaling, which contrasts with the regulation of other immune checkpoints like PD-L1.

To investigate whether YAP plays a role in modulating CD276 expression, we conducted experiments using EBV-positive AGS, GT38, and SNU-719 GC cell lines. Overexpression of YAP resulted in increased expression (Fig. 4F), while knockdown of YAP led to significant reduction in both YAP and CD276 levels (Fig. 4G, H). Notably, re-expression in YAP-knockdown cells rescued CD276 expression (Fig. 4I), confirming that YAP directly regulates CD276 expression.

To explore the molecular mechanisms underlying YAP-mediated regulation of CD276, we performed ChIP-seq experiments using Flag-tagged YAP1 (YAP1-Flag) in GT38 cells. Motif analysis revealed that DNA-binding motifs for the TEAD transcription factors, particularly TEAD3 and TEAD4, were enriched at YAP1 bound regions (Supplementary Fig. 8), suggesting that TEAD family members may play a role in YAP1-mediated regulation of CD276. RNA-seq data of EBVaGC further supported this hypothesis, as both TEAD3 and TEAD4 expression were significantly higher in the CD276-High group of EBVaGC tumors (*p* < 0.001) (Fig. 4J). Furthermore, low expression of TEAD3 or TEAD4 was strongly associated with reduced CD276 levels (*p* < 0.001) (Supplementary Fig. 9A). Consistent with these observations, correlation analysis revealed that CD276 expression was positively correlated with the expression of TEAD3 and TEAD4 (*r* = 0.416 and 0.437, *p* = 0.003 and 0.002, respectively), while no significant correlation was found with TEAD1 or TEAD2 (*p* = 0.018 and 0.262, respectively) (Fig. 4K).

To further investigate the role of TEAD in CD276 regulation, we treated YAP-overexpressing cells with verteporfin, an inhibitor that blocks the YAP-TEAD interaction, and two other YAP inhibitors, K-975 and dasatinib. The treatment significantly reduced CD276 expression and increased cytokines secretion of T cells in co-culture system, with verteporfin showing the most potent influence (Fig. 4L and Supplementary Fig. 9B–D). Given the evidence above that TEAD4 exhibited more significant statistical differences, knockdown of TEAD4 significantly decreased CD276 expression in EBVaGC cells (Fig. 4M). These data suggest that TEAD4, in concert with YAP, plays a pivotal role in the regulation of CD276.

**Fig. 5:**
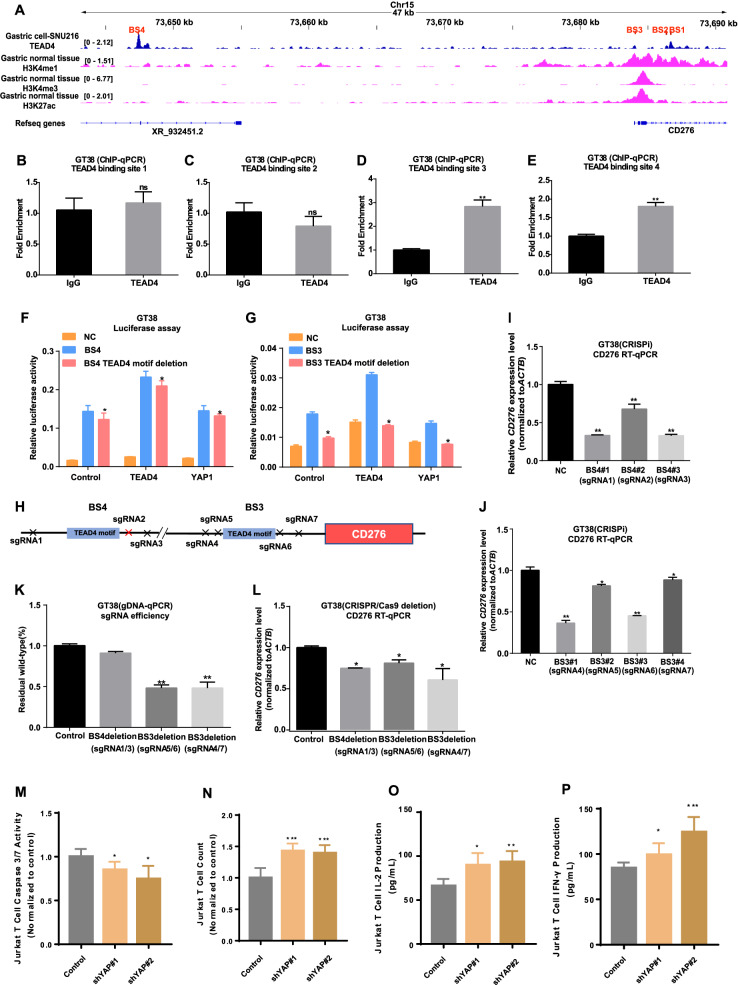
YAP-TEAD4-mediated transcriptional regulation of CD276. **A** ChIP-seq analysis of TEAD4 binding sites (BS1, BS2, BS3, BS4) in gastric cancer cells (SNU28). Active chromatin features in gastric specimens were identified via ChIP-seq for enhancer markers H3K27ac and H3K4me1 and promoter marker H3K4me3. **B–E** ChIP-qPCR analysis showing TEAD4 enrichment at BS3 and BS4 but not at BS1 or BS2. Data represents mean ± s.d. of three replicates. *P* values were calculated using a two-tailed Student’s *t*-test. **F–G** Functional validation of BS3 and BS4 using luciferase reporter assays. Wild-type or TEAD4-binding site mutants of BS3 and BS4 were cloned upstream of the luciferase gene, and activity was measured in GT38 cells. Relative luciferase activity was calculated as the ratio of firefly to Renilla luciferase activity. Data represent mean ± s.d. of three replicates. *P* values were calculated using a two-tailed Student’s *t*-test. **H** Schematic of the CRISPR strategy targeting the TEAD4 DNA motifs. **I**, **J** RT-qPCR analysis showing decreased CD276 expression upon CRISPR-mediated repression of BS3 and BS4. Expression levels were normalized to cells treated with non-targeting sgRNA (sg-NC). Data represent mean ± s.d. of three biologically independent experiments. *P* values were derived from a two-tailed *t*-test. **K** qPCR validation of CRISPR cutting efficiency. Primer pairs proximal to the PAM sequences of each sgRNA were used, with results normalized to the LP34 genomic locus. Fold changes were calculated relative to non-targeting sgRNA controls. *P* values were derived from a two-sided *t*-test. **L** RT-qPCR measurement of CD276 expression after partial genomic deletion of BS3 and BS4 using CRISPR/Cas9. Expression levels were normalized to sg-NC controls. Data represent mean ± s.d. of three biologically independent experiments. **M**, **N** In a co-culture system using activated Jurkat T cells and SNU-719 cells, YAP knockdown reduced apoptosis in Jurkat T cells and increased residual cancer cell numbers. **O**, **P** YAP knockdown significantly increased IFN-γ and IL-2 secretion in the co-culture system. *P* values were evaluated using a two-sided *t*-test. *P < 0.05, **P < 0.01.

To determine whether CD276 is a direct target of YAP/TEAD4, we performed a comprehensive analysis using the Cistrome DB [36], which houses over 60,000 processed ChIP-seq datasets. This analysis identified four TEAD4-binding peaks with active enhancer and promoter epigenetic marks (H3K4me1/3 and H3K27ac) in the *CD276* regulatory region in gastric cancer cells and normal stomach tissue (Fig. 5A). To validate these findings, we performed ChIP-qPCR assays, which confirmed the enrichment of TEAD4 at two specific sites, termed site 3 (BS3) and site 4 (BS4) (Fig. 5B–E).

To assess the transcriptional activity of TEAD4 binding sites, we constructed luciferase reporter plasmids containing the BS3 and BS4 sequences, both of which harbor TEAD4 motifs. The results showed that both BS3 and BS4 exhibited significant transcriptional activity, while mutations in the TEAD4 motifs, particularly within BS3, significantly reduced luciferase activity (Fig. 5F–G), confirming that these sites possess YAP1/TEAD4-mediated transcriptional activity.

Next, we utilized CRISPR interference (CRISPRi) and CRISPR/Cas9-mediated deletion to disrupt the TEAD4 binding motifs within BS3 and BS4, examining their impact on CD276 expression in GT38 cells (Fig. 5H). The CRISPRi assay showed a marked reduction in CD276 expression when either BS3 or BS4 was targeted (Fig. 5I, J and Supplementary Fig. 10). Similarly, deletion of BS3 or BS4 using CRISPR/Cas9 also led to a significant decrease in CD276 (Fig. 5K, L). These findings strongly suggest that YAP1/TEAD4 directly regulates CD276 transcription through these binding sites.

To confirm that YAP interacts with TEAD4 at the CD276 promoter, we conducted ChIP-qPCR assays in GT38 cells. Our results showed that YAP binds to the gene regulatory elements at BS3 and BS4 (Supplementary Fig. 11A, B), where TEAD4 also binds. Notably, CRISPR/Cas9-mediated deletion of BS3 or BS4 significantly reduced YAP1 enrichment at these sites (Supplementary Fig. 11C, D), further supporting that YAP1 co-occupies these regions with TEAD4 to regulate CD276 expression.

To validate the role of YAP as a key regulator of immune suppression, we utilized two YAP knockdown clones of the SNU719 cell lines in a co-culture system with Jurkat T cells for in vitro validation. YAP knockdown effectively reduced apoptosis in the Jurkat T cells (Fig. 5M, N) and significantly increased the secretion of IFN-γ and IL-2, indicative of enhanced T cell activity (Fig. 5O, P).

We further examined the role of YAP blockade in vivo using a xenograft tumor model in humanized immune mice. Following inoculation with the SNU719 gastric cancer cell line, mice received weekly treatments of either DMSO or verteporfin, a YAP-TEAD interaction inhibitor. YAP inhibition led to tumor suppression comparable to that observed with CD276 inhibition. Specifically, YAP blockade not only suppressed tumor growth (Supplementary Fig. 12A, B) but also significantly increased CD8+ T cell infiltration within the tumor microenvironment (Supplementary Fig. 12C).

Taken together, these findings underscore the pivotal role of YAP1/TEAD4 in regulating CD276 expression and immune evasion mechanisms in EBVaGC. Disruption of the YAP1/TEAD4 complex through CRISPR-mediated editing or pharmacological inhibition represents a promising strategy to enhance anti-tumor immune responses by increasing CD8+ T cell infiltration and reducing tumor-mediated immunosuppression.

### EBV-encoded EBNA1 mediates YAP pathway activation and CD276 overexpression in EBVaGC

**Fig. 6:**
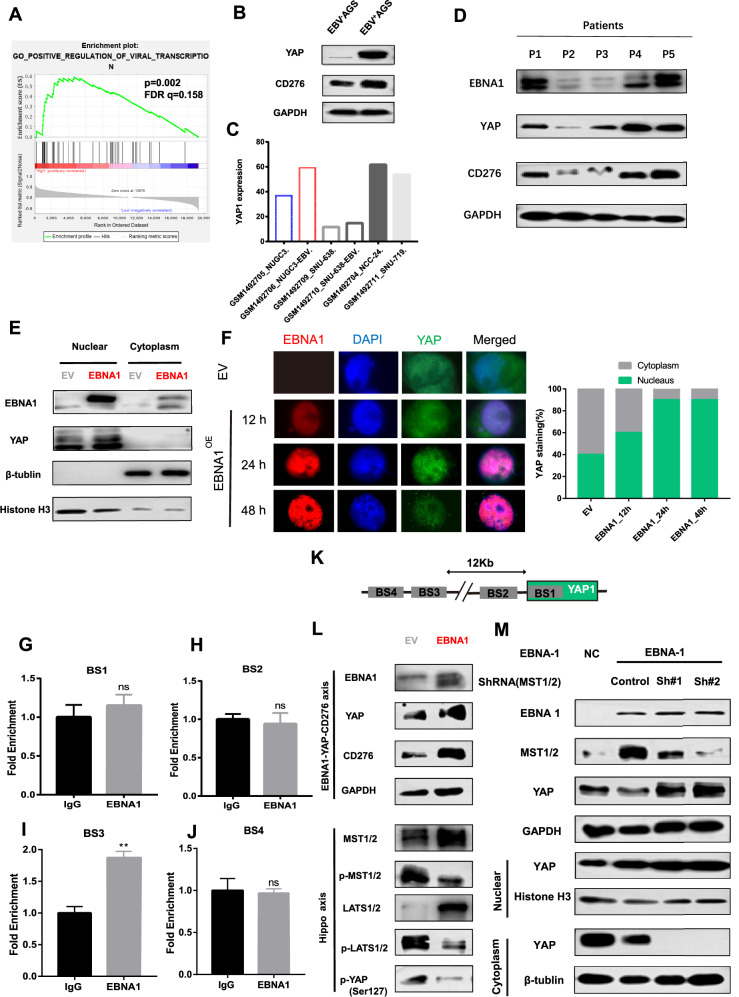
EBV-encoded EBNA1 activates YAP through the Hippo signaling axis. **A** Gene set enrichment analysis (GSEA) of the “positive regulation of viral transcription” GO gene set, showing significant enrichment in the CD276-High group. **B** YAP and CD276 expression levels before and after EBV infection in AGS cells. **C** Increased YAP1 transcriptional levels in NUGC3 and SNU-638 cells upon EBV infection, as well as relatively higher YAP1 mRNA levels in naturally EBV-positive cell lines (NCC-24 and SNU-719). Data obtained from transcriptomic analysis of human gastric cancer cell lines naturally infected with EBV (GSE60873). **D** Western blot analysis of prospectively collected tissue samples demonstrating a positive correlation between EBNA1 and YAP protein expression levels. **E** Ectopic expression of EBNA1 promotes YAP protein levels, with notable upregulation observed predominantly in the nucleus. **F** Time-course immunofluorescence analysis of AGS cells overexpressing EBNA1, showing YAP localization using an anti-YAP antibody. EBNA1 overexpression induced progressive YAP nuclear accumulation, with maximal nuclear/cytoplasmic YAP ratio at 24 h post-transfection. **G–J** ChIP-qPCR validation of EBNA1 binding sites within the YAP DNA region in EBNA1-overexpressing GT38 cells. **K** Schematic representation of EBNA1 binding sites. **L** Western blot analysis showing the effects of EBNA1 overexpression on the YAP-CD276 axis and the Hippo signaling axis. **M** MST knockdown enhances YAP nuclear translocation, amplifying its functional activity.

To investigate the potential role of EBV in activating the YAP-CD276 regulatory axis in EBVaGC, we conducted gene set enrichment analysis (GSEA) of the CD276-High group from our EBVaGC gene expression dataset. We observed significant enrichment in the “positive regulation of viral transcription” Gene Ontology (GO) pathway (Fig. 6A), along with differential expression of virus-related genes between the CD276-High and CD276-Low groups (Supplementary Fig. 2), suggesting that EBV infection could directly impact the activation of the YAP-CD276 signaling pathway. Indeed, infection of AGS cells with EBV led to a notable increase in YAP expression (Fig. 6B). This finding was consistent with results from a publicly available dataset (GSE60873), where EBV infection also induced YAP expression in NUGC3 and SNU-638 cell lines (Fig. 6C).

To further explore the link between EBV infection and YAP activation, we analyzed RNA-seq data from our EBVaGC cohort, focusing on the expression of EBV-encoded proteins and their correlation with YAP. Notably, EBV-encoded transcripts correlated with increased YAP1 expression, with EBNA1 showing the strongest, though marginal, statistical association (p = 0.058) (Supplementary Fig. 13). Western blot analysis of tissue samples confirmed a positive correlation between EBNA1, YAP, and CD276 protein expression levels (Fig. 6D). In AGS cells transfected with an EBNA1 plasmid, YAP expression was significantly upregulated (Fig. 6E), and importantly, YAP was predominantly localized in the nucleus (Fig. 6E, F), suggesting that EBNA1 activates YAP at both the transcriptional and protein translocation levels.

YAP, a central regulator in the Hippo pathway, primarily mediates its effects by transcriptionally regulating target gene [29]. EBNA1 is known to bind directly to human DNA, acting as a transcription factor, and our data suggest that it may influence YAP expression. In a ChIP-qPCR assay conducted in EBNA1-overexpressing GT38 cells, we found that EBNA1 binding was significantly enriched at a specific regulatory site, designated BS3, within the YAP gene (Fig. 6G–K), suggesting a direct role for EBNA1 in modulating YAP transcription.

At the protein level, YAP activation involves dephosphorylation and nuclear translocation, processes regulated by upstream kinase such as MST1/2 and LATS1/2 in the Hippo pathway. Our data revealed that EBNA1 transfection not only upregulated the expression of YAP and CD276 but also increased the levels of MST1/2 and LATS1/2 while facilitating their dephosphorylation (Fig. 6L). Furthermore, knockdown of MST resulted in enhanced nuclear translocation of YAP (Fig. 6M). These findings suggest that EBNA1-mediated activation of the YAP pathway leads to CD276 overexpression in EBVaGC, highlighting a YAP-dependent mechanism of immune evasion.

### Higher CD276 expression impairs the efficacy of anti-PD1 treatment in EBVaGC patients

The development of “cold tumors”, which are characterized by low immune cell infiltration, poses a significant challenge to the effectiveness of cancer immunotherapies, particularly immune checkpoint inhibitors. To assess the impact of CD276-mediated immune suppression on the response to immunotherapy, we conducted a clinical evaluation of EBVaGC patients undergoing anti-PD1 treatment.

**Fig. 7:**
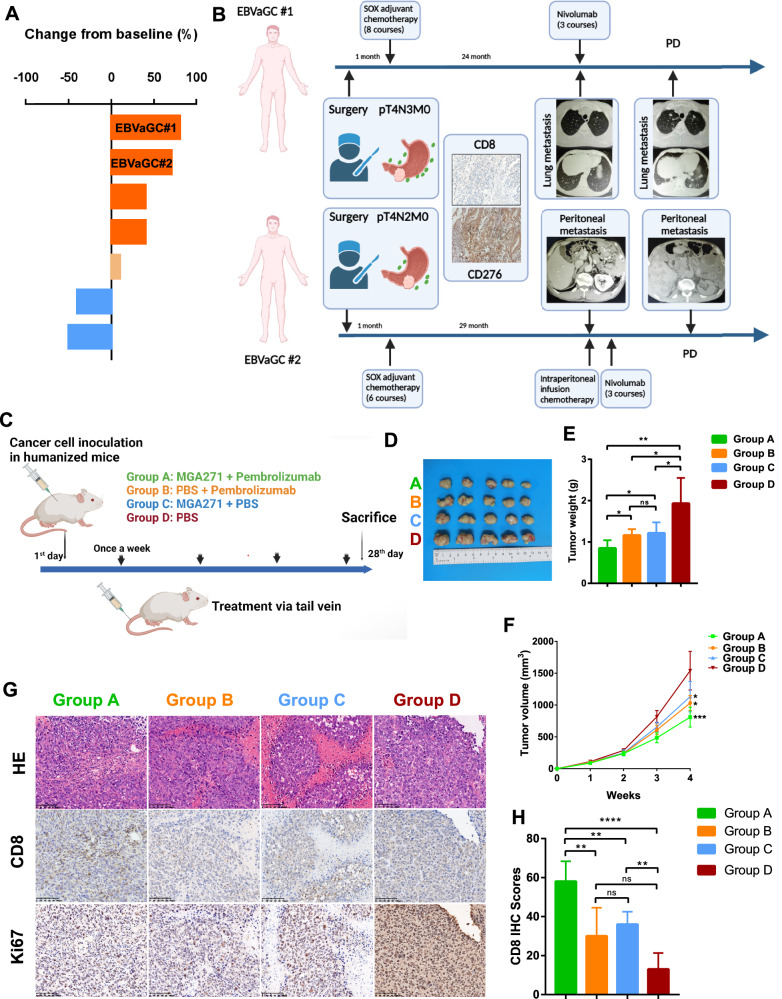
Efficacy of anti-PD1 treatment in CD276-High EBVaGC. **A** Clinical evaluation of anti-PD1 therapy in seven patients with EBVaGC. **B** Two patients (EBVaGC#1 and #2) exhibited significant disease progression after three courses of anti-PD1 therapy, with high CD276 expression levels. **C** Schematic representation of the xenograft model used for in vivo validation of anti-PD1 treatment efficacy. **D** Tumor masses excised from xenograft models using SNU719 cells in humanized mice after 4 weeks of treatment. **E** Comparison of tumor weights across treatment groups: the heaviest tumors were observed in the placebo group (Group D), while the lightest were found in the combination treatment group (Group A). **F** Tumor volume growth curves for each treatment group. **G** Histological analysis of paraffin-embedded mouse tumors, including H&E, CD8, and Ki67 staining. Results are presented as successive sections of the same tumor site. **H** Comparison of CD8 IHC scores among different treatment groups.

We recruited seven EBVaGC patients who received nivolumab (anti-PD1) therapy (Fig. 7A), and the treatment outcomes were categorized according to Response Evaluation Criteria In Solid Tumors (RECIST) as progressive disease (PD), stable disease (SD), partial response (PR), or complete response (CR). Of the seven patients, only 29% (2/7) achieved a partial response (PR), while one patient had stable disease (SD), and the remaining four patients experienced disease progression (PD). Notably, EBVaGC#1 and EBVaGC#2 showed the most severe progression.

Specifically, EBVaGC#1, initially diagnosed with a pT4N3M0 tumor, underwent surgery followed by eight cycles of SOX adjuvant chemotherapy. After 24 months, lung metastasis developed, and the patient received three courses of SOX + nivolumab. Despite this, disease progression was evident. EBVaGC#2 underwent surgery and six cycles of SOX adjuvant chemotherapy for a pT4N2M0 tumor. This patient developed peritoneal metastasis after 29 months and was treated with intraperitoneal infusion chemotherapy in combination with nivolumab. However, progression was observed after three courses of anti-PD1 therapy.

Histological analysis of tumor biopsies from these two patients revealed a marked reduction in CD8+ T cell infiltration and elevated CD276 expression (Fig. 7B). These findings suggest that the high expression of CD276 may mediate immune evasion by depleting CD8+ T cells, thereby impairing the efficacy of anti-PD1 treatment. This indicates that CD276 may contribute to the establishment of an immunosuppressive microenvironment that limits the effectiveness of PD-1 blockade.

### Combination therapy of targeting CD276 and PD1 in EBVaGC xenograft models

To evaluate the therapeutic potential of targeting CD276 and its combination with PD1 inhibition, we established xenograft tumors using the SNU719 cells in humanized mice (Fig. 7C). Mice were randomly assigned to four treatment groups. Group A was subject to combination therapy of CD276 targeting (MGA271) and PD1 inhibition (pembrolizumab). Group B and Group C received MGA271 or pembrolizumab monotherapy, respectively, plus PBS, while Group D treated only with PBS as negative control group.

Therapies were administered weekly for 4 weeks following tumor inoculation. Tumor growth was measured regularly, and the final tumor sizes were recorded (Fig. 7D–H). The combination therapy group (Group A) exhibited the smallest tumor size compared to the other groups, indicating superior tumor control (Fig. 7D, E). The negative control group (Group D), which received PBS treatment only, showed the largest tumor size, followed by the monotherapy groups (Groups B and C). Importantly, while both CD276 targeting and PD1 inhibition demonstrated anti-tumor effects compared to the control group, no significant difference was observed between the two monotherapy groups (Fig. 7E, F). These results suggest that targeting CD276 in combination with PD1 inhibition significantly enhances the therapeutic compared to either therapy alone.

Further analysis through IHC revealed increased CD8+ T cell infiltration in Group A, which likely contributed to the improved anti-tumor efficacy (Fig. 7G, H). In contrast, Group D, the negative control group, exhibited strong Ki67 staining, indicating high proliferative activity in the absence of treatment. These findings suggest that the combination therapy not only suppresses tumor growth but also promotes a more robust anti-tumor immune response.

## Discussion

**Fig. 8:**
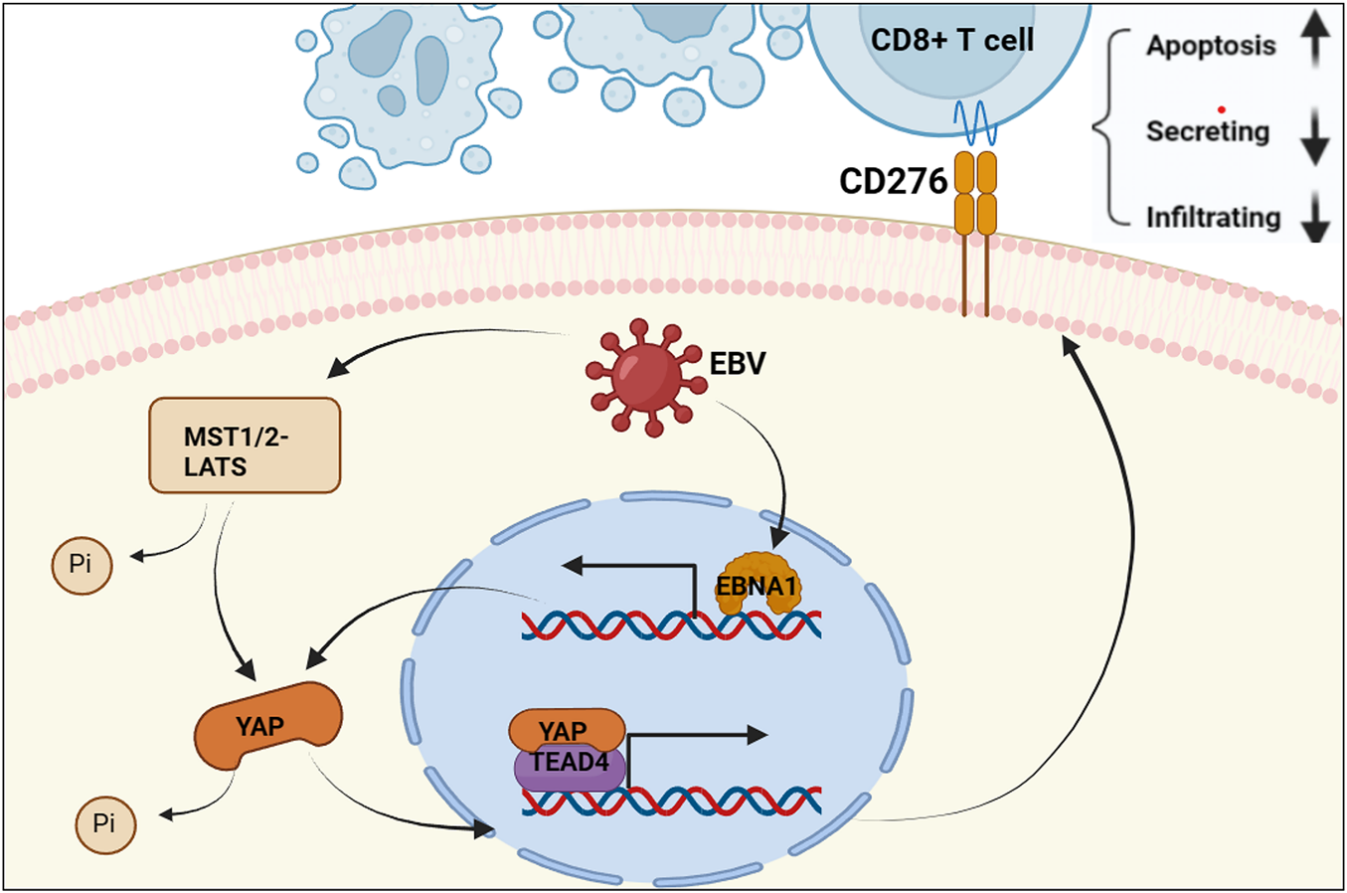
Schematic representation of the proposed mechanism. The proposed mechanism illustrates how EBNA1-stimulated YAP signaling facilitates immune escape in EBV-associated gastric cancer (EBVaGC). Activation of YAP signaling by EBNA1 enhances the chromatin occupancy of YAP/TEAD4 complexes at the regulatory regions of the CD276 gene, leading to its transcriptional upregulation. The resultant elevated CD276 expression drives immune evasion within the tumor microenvironment, emphasizing a pivotal pathway in EBVaGC progression. The figure is created with BioRender.com.

Our study sheds light on the immune-resistant phenotype of EBVaGC, characterized by low immune cell infiltration and low PD-L1 expression, which together contribute to the formation of “cold” tumors that are resistant to current immunotherapy approaches. Through comprehensive transcriptome and IHC profiling, we identified CD276 as a pivotal immune regulatory factor that significantly reduces lymphocyte infiltration within EBVaGC. Additionally, we uncovered a novel role of the EBV-encoded protein EBNA1 in activating the YAP pathway, a critical regulator of CD276 expression at the transcriptional level (Fig. 8). This mechanistic insight into immune evasion offers potential for novel therapeutic strategies to overcome this resistance.

Recent studies have evolved our understanding of CD276 [37], shifting its role from a presumed co-stimulatory molecule in antigen recognition to an immune co-suppressive molecule. This nuanced perspective aligns with our findings, which establish CD276 as an immunosuppressive protein that restricts immune cell infiltration in EBVaGC. RNA sequencing and IHC analyses reveal that CD276 not only forms a barrier at the tumor’s invasive margin, obstructing T cell infiltration, but also promotes T cell apoptosis and suppresses their secretory activity. These effects collectively weaken immune responses, correlating with poor prognoses and resistance to anti-PD1 therapies, thus supporting CD276 as an immune suppressor in EBVaGC.

Anti-PD1 immunotherapy, which relies on robust T cell infiltration within the tumor microenvironment, is less effective in tumors with limited lymphocytic infiltration [38, 39]. Our findings suggest that CD276-mediated T cell apoptosis and reduced secretion are key contributors to inadequate T cell presence for effective immunotherapy. Notably, two EBVaGC patients with high CD276 expression in our study exhibited rapid progression following nivolumab treatment, a pattern also observed in lung cancer patients where CD276 positivity was associated with poor response to anti-PD1 therapies [40]. Given that CD276 blockade has been shown to enhance CD8+ T cell infiltration in preclinical lung cancer models [40], our findings underscore the potential of combining CD276 inhibitors with PD-1 pathway inhibitors as a therapeutic approach for EBVaGC.

Unlike other immune checkpoints, such as PD-L1, which are often associated with immune activation, our study demonstrates that CD276 suppresses immunity independently. PD-L1 expression, generally induced by inflammatory cytokines like IFN-γ, serves as a feedback mechanism to immune activation [39, 41–43]. However, CD276 in EBVaGC is not be upregulated by IFN-γ, suggesting a distinct regulatory pathway. Tumors with high CD276 expression lack markers of immune activation, suggesting that its expression may be driven by intrinsic tumor characteristics rather than an immune response. This unique expression pattern, also observed in non-small-cell lung cancer (NSCLCs) [44], underscoring the potential for combinatorial therapeutic strategies that target CD276 alongside conventional immunotherapies.

Our findings also define the role of the Hippo-YAP pathway in CD276 regulation. We identify the Hippo pathway, particularly the YAP1-TEAD4 complex, as a critical regulator of CD276 in EBVaGC. The strong co-expression of YAP1 and CD276 suggests a transcriptional regulation axis that could represent a new therapeutic target. Targeting the YAP-TEAD4-CD276 pathway may convert “cold” tumors into “hot” ones, facilitating immune infiltration by increasing T and B cells while reducing immunosuppressive cell populations such as MDSCs, M2 macrophages, and Treg cells [45–47]. Given the known role for YAP in modulating genes crucial to tumor growth, a therapeutic approach targeting both the YAP-TEAD4-CD276 axis and PD-1 could offer substantial clinical benefits.

We further explored the role of EBV in immune evasion through the YAP pathway. EBV in modulating oncogenic pathways has been well-documented, with proteins such as LMP previously shown to regulate YAP/TAZ activity [29]. Our study reveals that EBNA1 overexpression can directly activate the YAP pathway, both by enhancing YAP transcription and through the MST-LATS-YAP dephosphorylation pathway. Additionally, EBNA1 binds to the oriP sequence [48], facilitating the production of viral proteins and reinforcing YAP activation. This mechanism enables the tumor to evade immune surveillance while simultaneously shielding the virus from immune attacks, establishing a robust symbiotic relationship that supports both viral persistence and tumor progression.

In conclusion, our study identifies CD276 as a crucial factor in the immune evasion of EBVaGC, regulated by the viral activation of the YAP/TEAD4 pathway. These findings suggest that targeting CD276 in combination with PD-1 inhibitors may enhance immunotherapy response. Our insights into EBVaGC immune regulation open new avenues for therapeutic strategies that address immune suppression within the tumor microenvironment, with the potential for developing innovative treatments to improve patient outcomes.

## Materials and methods

### Patients and samples

From 2014 to 2018, a total of 50 EBVaGC patients who underwent surgical resection at Sun Yat-sen University Cancer Center (SYSUCC) were included in this study. The inclusion criteria were as follows: (1) underwent curative gastrectomy for gastric adenocarcinoma; (2) pathologically diagnosed EBVaGC; (3) no preoperative systemic treatment; and (4) no other malignant tumors. EBV-encoded RNA (EBER)-in situ hybridization (EBER-ISH) and Hematoxylin and Eosin (H&E) staining were performed and reassessed by at least two independent pathologists to determine the diagnosis of EBVaGC. Clinical and pathological data included age, sex, tumor location, Lauren type and stage (Supplementary Table 1). In addition, we obtained RNA-seq data from The Cancer Genome Atlas (TCGA) cohort as an external validation cohort (*n* = 27).

### Transcriptome sequencing and bioinformatics processing

Total RNA was extracted from frozen tumor tissues and paired normal specimens using the RNeasy Mini Kit (QIAGEN, Valencia, CA). The library was prepared according to the manufacturer’s instructions and was subsequently subjected to high-throughput sequencing using a HiSeq X sequencer (Illumina HiSeq 2000 platform). More detailed information about data processing and the fragments per kilobase of transcript per million (FPKM) values generated was described previously [15].

The FPKM expression matrix was processed with Student’s *t*-test to identify differentially expressed genes (DEGs) across two EBVaGC groups: CD276-High vs. CD276-Low. Kyoto Encyclopedia of Genes and Genomes (KEGG) analysis was carried out with the R package “clusterProfiler” [49] on the DEGs between the two groups. Gene set enrichment analysis (GSEA) [49] was performed by the GSEA program from the Broad Institute. One thousand random sample permutations were carried out, and the significance threshold was set at NES absolute value > 1, NOM *p* value < 0.05, and FDR *q* value < 0.05.

The R package “MCP-counter” [32] was applied to calculate the absolute abundance scores for eight major immune cell types (CD3^+^ T cells, CD8^+^ T cells, cytotoxic lymphocytes, natural killer (NK) cells, B lymphocytes, monocytic lineage cells, myeloid dendritic cells, and neutrophils). The gene expression matrix of 50 EBVaGC patients was used as the input data to determine immune infiltration in the TME.

A custom script was used for virus quantification. The transcripts per million (TPM) value of the viral transcript was obtained from the StringTie results. The expression of EBV-encoded transcripts was obtained based on each specific sequence.

### Immunohistochemistry (IHC) analysis and evaluation

H&E staining and IHC staining were performed according to protocols detailed previously [15]. The primary antibodies and peroxidase (HRP)-conjugated secondary antibodies are shown in Supplementary Table S2. CD3, CD8, and FOXP3 were used to mark CD3- and CD8-positive T cells and Treg cells, respectively, while CD68 and CD163 were used to mark M1 and M2 cells, respectively. Positive staining was measured at the center of the tumor (CT) and at the invasive margin (IM). Specifically, the staining score at the CT was defined as the percentage of mononuclear inflammatory cells within an intraepithelial tumor cell nest that were in direct contact with tumor cells. Meanwhile, the staining score at the IM was defined as the percentage of tumor stroma of invasive carcinoma occupied by mononuclear inflammatory cells.

### Cell culture and reagents

The human gastric cancer AGS and EBV^+^AGS cell lines were obtained from Dr. Musheng Zen (Sun Yat-sen University Cancer Center). The cell line was cultured in DMEM (BasalMedia, L110) according to standard protocols. Two EBV-positive cell lines, GT38 and SNU-719, were obtained from the Cell Bank of the Chinese Academy of Sciences (Shanghai, China) and were maintained in RPMI 1640 medium (BasalMedia, L210). All media were supplemented with 10% fetal bovine serum (Gibco, 10, 270-106) and 1% penicillin–streptomycin (BasalMedia, S110B), and the cells were not passaged more than six times from collection to use.

### Expression vectors, plasmid transfection, and lentiviral infection

YAP1 (NM_001282100), CD276 (NM_001024736), and EBNA1 (YP_401677.1) cDNAs were obtained from GeneChem (Shanghai, China) and subcloned into the Ubi-MCS-3FLAG-SV40-EGFP-IRES-puromycin vector (GV358) and CMV enhancer-MCS-3FLAG-EF1a-ZsGreen1-T2A-puromycin vector (GV730) to generate the expression vector. Human YAP1 short hairpin RNAs (shRNAs) in U6-MCS-Ubiquitin-Cherry-IRES-puromycin (GV248) were also purchased from GeneChem.

To generate stable cell lines, HEK293T cells were transfected with each lentivirus expression vector and packaging plasmid mix using polyethyleneimine (PEI) DNA transfection reagent. The supernatant containing viruses was collected 48 h after transfection, filtered, and used to infect target cells in the presence of 10 μg/ml polybrene (Sigma–Aldrich, H9268) prior to drug selection with 1–2 μg/ml puromycin for one week. Overexpression and knockdown (KD) efficiency were validated by immunoblotting after transfection.

### Co-culture system and T cell assay

Jurkat T cell was treated with 1 μg/mL phytohaemagglutinin (PHA) and 50 ng/mL phorbol 12-myristate 13-acetate (PMA) for 2 days of activation. Following overexpressing CD276 in SNU-719 cell line, cancer cells were plated into 12-well plate, and activated Jurkat T cells were added to each well at a ratio of 1:10. After 24 hours of co-culture, T cells were collected for counting and apoptosis assay using Caspase 3/7 Activity Apoptosis Assay Kit (Sangong Biotech, E607103-0200). Moreover, supernatant was collected for IL-2 and IFN-γ production assay using ELISA kits from Absin according to the manufacturer’s instructions.

### Western blot (WB) analysis

Cells were lysed in Pierce T-PER® Tissue Protein Extraction Reagent (Thermo Fisher Scientific, Inc.) with protease and phosphatase inhibitors (Bimake, B14001, B15001A + B). The lysates were centrifuged at 12,000 rpm for 15 min, supernatants were collected, and a bicinchoninic (BCA) protein assay kit (Solarbio, Beijing, China, PC0020) was used to determine protein concentrations. A total of 20–30 μg of protein was separated by SDS–PAGE and transferred to PVDF membranes (Millipore, IPVH00010, ISEQ00010). The primary and secondary antibodies are detailed in Supplementary Table S2. ImageJ was used to quantify the immunoblotting results by protein band densities.

### Immunofluorescent staining

Immunofluorescent staining was carried out as described previously [15]. Briefly, the adherent cells after drug treatment were washed in PBS, fixed in 4% paraformaldehyde, permeabilized in 0.1% Triton X-100, and blocked in 1% bovine serum albumin (BSA) in PBS. The cells were incubated with primary antibody in 1% BSA at 4 °C overnight, washed three times in PBS for five minutes each, and then incubated with the appropriate secondary antibody (Jackson ImmunoResearch, 115-095-003/111-585-003). DNA staining was performed using ProLong™ Gold Antifade Mountant with DAPI (Invitrogen, P36931). Leica SP5 confocal laser scanning microscopy (Leica Microsystems, Buffalo Grove, USA) was used for immunofluorescence photography.

### ChIP-sequencing and ChIP-qPCR

ChIP-seq assay was performed as described previously [50]. In brief, GT38 cells were transfected with FLAG-YAP1 overexpression construct using polyethyleneimine (PEI) DNA transfection reagent, and after 48 h were cross-linked with 1% formaldehyde for 10 min, followed by 5 min of 125 mM glycine treatment to stop the reaction. Cells were washed twice with cold PBS and lysed with hypotonic lysis buffer (10 mM KCl, 20 mM Tris-Cl, PH 8.0, 2 mM DTT, 10% glycerol, and Complete protease inhibitor cocktail (Roche)) to extract nuclei. The nuclei were suspended in SDS lysis buffer (50 mM Tris-HCl, with 0.1%SDS, 10 mM EDTA) supplemented with protease inhibitors. Chromatin was sonicated by Bioruptor® Pico (pulse: 30 s on/30 s off; 5 cycles) to generate 100–300 bp fragments at 4 °C and immunoprecipitated with 6 µg Flag (sigma, F1804) or control IgG (Santa Cruz, sc-2025) antibodies premixed with 35 µl Dynabeads protein G slurry (Invitrogen, 10004D). Next, the chromatin-antibody-beads complex was washed once with low salt wash buffer (0.1% SDS, 1% Triton X-100, 2 mM EDTA, 20 mM Tris-HCl, PH = 8.1, 150 mM NaCl) and once with high salt wash buffer (0.1% SDS, 1% Triton X-100, 2 mM EDTA, 20 mM Tris-HCl, PH = 8.1, 500 mM NaCl), and followed by washing once with LiCl wash buffer(250 mM LiCl, 1% NP-40, 1% Deoxycholate, 1 mM EDTA, 10 mM Tris-HCl, PH = 8.1) and twice with TE buffer (10 mM Tris-HCl, PH = 8.1, 1 mM EDTA, 50 mM NaCl). Then, the 100 μl extraction buffer (50 mM Tris-HCl, PH = 8.1, 10 mM EDTA, 1% SDS) was added to elute DNA-protein complex from the antibody/protein G magnetic beads for 30 min at 65 °C with gentle vortexing (1200 rpm) and incubated with 0.3 M NaCl and proteinase K to reverse the cross-links at 65 °C overnight. The enrichment DNA was extracted by Mini-Elute PCR purification kit (Qiagen, 28006). ChIP-seq libraries were prepared using NEBNext DNA Ultra II library prep kit and NEBNext Multiplex Oligos for Illumina (96 Unique Dual Index Primer Pairs), and sequenced by Illumina NovaSeq.

ChIP-seq data were aligned to hg38 human genome assembly with Bowtie2 [51]. We used SAMtools [52] to sort and index the aligned reads, and MACS2 [53] to calculate signal per million reads (SPMR) and call significant ChIP-seq peaks (*q* value < 0.05). DNA Motif enrichment analysis was performed using HOMER software [54].

For ChIP-qPCR assays, we utilized the Flag and TEAD4 ChIP-seq data^60^ to design primers targeting YAP1/TEAD4 binding sites in CD276 promoter and enhancer regions. The data were normalized to the control regions, then relative enrichment of the TEAD4 antibody (Santa Cruz, sc-390578×) at the target regions was determined by compared with the background (IgG control). All the qPCR primers are listed in Supplementary Table 3.

### Construction of reporter vector and luciferase reporter assay

To evaluate the transcriptional activity of TEAD4 binding site 3 (BS3) and 4 (BS4), DNA fragments surrounding the binding sites were amplified from genomic DNA using the primer pair BS3-F/R (*Kpn I*/*Hind III*) and BS4-F/R(*Kpn I*/*Hind III*) and cloned into pGL4.23 (Promega) and pGL4.10 (Promega). For construction of the TEAD4 motif (13 bp) null mutants, PCR reactions were performed in order to amplify sequences upstream and downstream of the intended deletion. The upstream fragments were amplified from BS3 and BS4 constructs using primer pair BS3-homF/mutR and BS4-homF/mutR, while the downstream fragments were amplified with primer pair BS3-mutF/homR and BS4-mutF/homR. The two PCR products were cloned into *Kpn I*/*Hind III-*digested pGL4.23 and pGL4.10, respectively, yielding BS3m and BS4m luciferase reporter constructs. For overexpression construct, TEAD4 cDNA was amplified using primer pairs TEAD4-F/R (BamHⅠ/NotⅠ) and subcloned into pCDNA3.1 vector with V5 tagged. All constructs were confirmed by Sanger sequencing.

0.4 µg of luciferase reporter constructs and 0.4 µg of either overexpression plasmid or empty vector were transfected into GT38 cells using polyethyleneimine (PEI) DNA transfection reagent according to the manufacturer’s instruction. The internal Renilla control plasmid pGL4.75 (4 ng) was co-transfected to standardize transfection efficiency. After 48 h, the luciferase activity was determined using the Dual Luciferase Reporter Assay System (Promega). The experiments were performed in triplicate. Data are presented as firefly/*Renilla* luciferase activity. All the PCR primers are listed in Supplementary Table 3.

### CRISPR-mediated transcriptional repression and genomic deletion of TEAD4 binding site

We first established the GT38 cells stably expressing the dCas9-KRAB and Cas9, respectively. In detail, lentivirus was produced by transfecting 293 T cells with Lenti-Cas9-Blast and dCas9-KRAB (Addgene, 154472) expression plasmid together with the packaging plasmids psPAX2 (Addgene, 12260) and pMD.2G (Addgene, 12259) using polyethyleneimine (PEI) DNA transfection reagent. The virus-containing supernatant was collected 48 h and 72 h following transfection and passed through 0.45 µm filter. We then infected GT38 cells with the lentivirus in the presence of 8 µg/ml polybrene, and the medium was replaced with fresh medium after 24 h. The infected GT38 cells were selected with blasticidin (1 µg/ml) until uninfected cells died completely.

20-nt single-guide RNA (sgRNA) were designed based on the genomic sequences close to TEAD4 binding site 3 and 4 and evaluated for potential off-target activity using the CRISPR Design tool (http://crispr.mit.edu/). For CRISPR interference (CRISPRi), sgRNAs were subcloned into pgRNA (Addgene, 44248) according to the protocol [55]. Then we infected the dCas9-KRAB cells with pgRNA carrying either negative control or target sgRNA, and selected the cells with puromycin (1 µg/ml) for 2 days. For CRISPR-mediated deletion of TEAD4 motif identified in the BS3 and BS4 region, the sgRNA sequences were synthesized as single-stranded oligonucleotides. The Cas9-expressing GT38 cells were seeded at 5 × 10^4^ per well in 24-well plates and allowed to attach for 24 h, and transfected with 0.5 µg sgRNA oligonucleotides using polyethyleneimine (PEI) DNA transfection reagent. Total RNA and genomic DNA were isolated by TRIzol Reagent (Invitrogen, 15596026) according to the manufacturer’s instruction after 48 h transfection. To validate CRISPR/Cas9 mediated cutting efficiency, DNA fragments (~800 bp) containing sgRNA binding region were PCR amplified from genomic DNA followed by Sanger sequencing. In addition, for qPCR-based quantification of the efficiency, pairs of primers proximal to PAM sequence to each sgRNA were designed. Relative efficiency for genomic DNA amplification was normalized to the intron region of gene *LIPC*, compared to sgRNA targeting the intergenic region [56]. To determine expression of CD276, cDNA was synthesized using PrimeScript^TM^ RT Master Mix (TaKaRa, RR036Q) from 2 µg RNA. The TB Green Premix Ex TaqII (TaKaRa, RR820A) was used in quantitative RT-PCR reaction. sgRNA targeting an intergenic region was used as negative control. *CD276* gene expression was normalized to that *GAPDH*, and ΔΔCt values were calculated using sgRNA targeting the intergenic region as the control sample. All qPCR primers and sgRNA sequences are listed in Supplementary Table 3.

### Humanized mouse xenograft models

PERCOLL from Biosharp was diluted to a concentration of 60% and a density of 1.077 g/ml for lymphocyte separation. Peripheral blood was obtained from healthy donors after signing informed consent. Mixed with equal volume of PBS, peripheral blood was added to the top of diluted PERCOLL and centrifuged to separate layers. The buffy coat was extracted, removed residual red blood cells using lysate, washed, and counted to obtain human peripheral blood mononuclear cells (PBMCs).

The 5-week-old male NOD-SCID IL2Rγ^null^ mice were infused PBMCs (1 × 107 per mouse) via tail vein to build immune humanized models. Then, SNU-719 cells (5 × 106 per mouse) were harvested and resuspended in 50 μl of PBS and 50 μl of Corning Matrigel (BD Biocoat, USA, 354248). The mixture was inoculated subcutaneously on the next day. During the tumor growth, mice blood was collected via submandibular vein for flow cytometry every week to examine the efficiency of immune reconstitution. In addition, mice were weighed weekly to monitor the occurrence of severe xeno-GvHD. Tumor volumes were measured once a week and calculated as follows: length × width^2^/2. After 4 weeks of tumor growth, mice were sacrificed and xenografts were obtained for making formalin fixed and paraffin-embedded sections. IHC was performed to evaluate the immune infiltration in the mice tumor. No randomization or blinding was applied in the animal experiments.

### Flow cytometry

Fifteen freshly resected tumor tissues were obtained from Fudan University Shanghai Cancer Center to examine the CD276 and the functional molecules of CD8+ cells, including PRF-1, GZMB, and IFN-γ. Signed informed consent was obtained from each patient. The antibodies used in flow cytometry are listed in Supplementary table 2.

### Statistics

Potential associations between CD276 expression and clinical characteristics were assessed for significance using the χ2 test or Fisher’s exact test. Kaplan–Meier curves with log-rank tests were used to evaluate the survival outcomes. Intergroup differences in gene expression and IHC scores were assessed for significance using the two-tailed Student’s *t*-test, and correlation coefficients were calculated using the Pearson test. In addition to the algorithms described above, all statistical analyses were performed in R version 3.4.1 software (R, CA, USA), GraphPad Prism 7.0 (GraphPad Software, Inc.), and SPSS22.0 (SPSS Inc., Chicago, IL). *P* value of <0.05 was considered statistically significant.

## Supplementary information

Supplementary figures

Supplementary table 1-2

Supplementary table 3

Supplementary material legends

original blots image

## Data Availability

The data that support the findings of current study are available from the corresponding author upon reasonable request.
