# Supplementary material for: An EBNA1-YAP signaling axis drives immune escape through CD276 in EBV-associated gastric cancer
Source: Cell Death Dis. 2025 Dec 19;17(1):118. doi: 10.1038/s41419-025-08251-2 (PMC12847768; doi:10.1038/s41419-025-08251-2)
Supplement: Supplementary file 1 — Supplementary figures [file 41419_2025_8251_MOESM1_ESM.pdf]

Supplementary Figure 1-13

Supplementary Figure 1

A

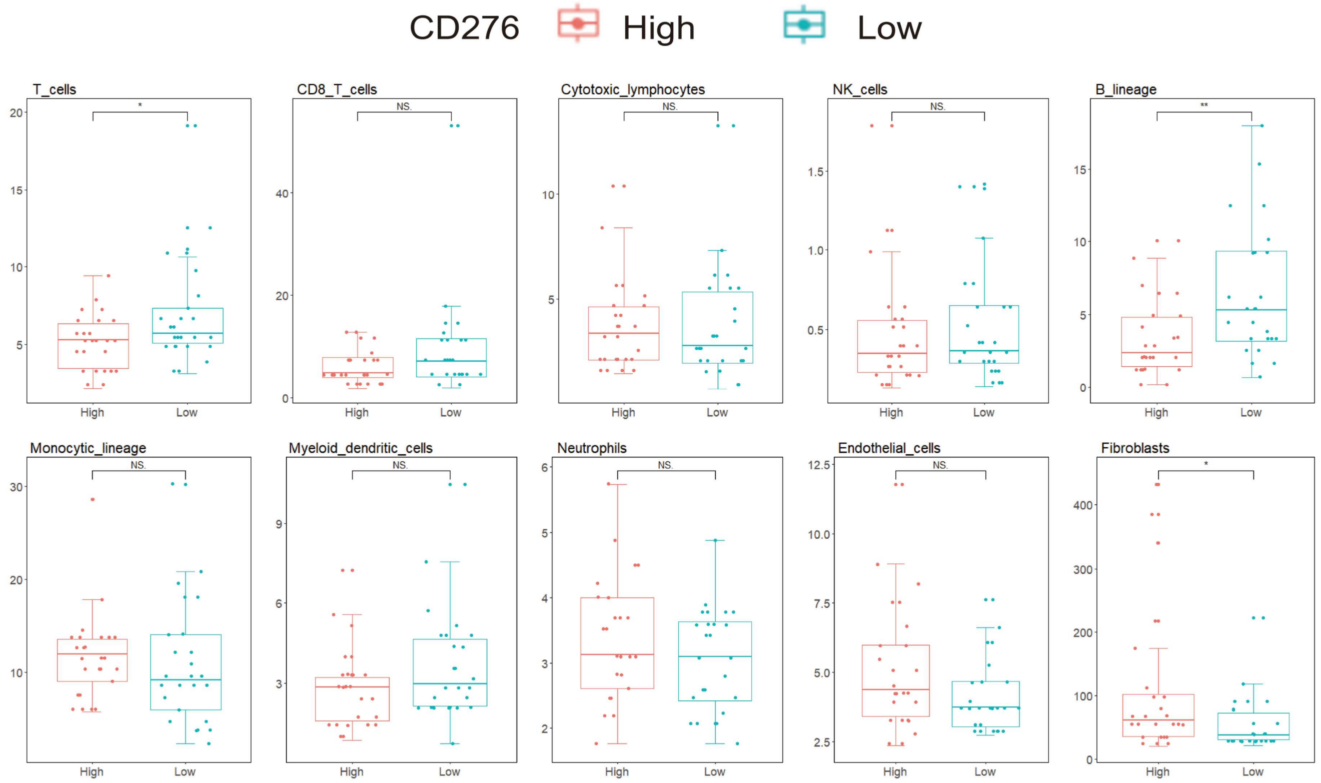

B

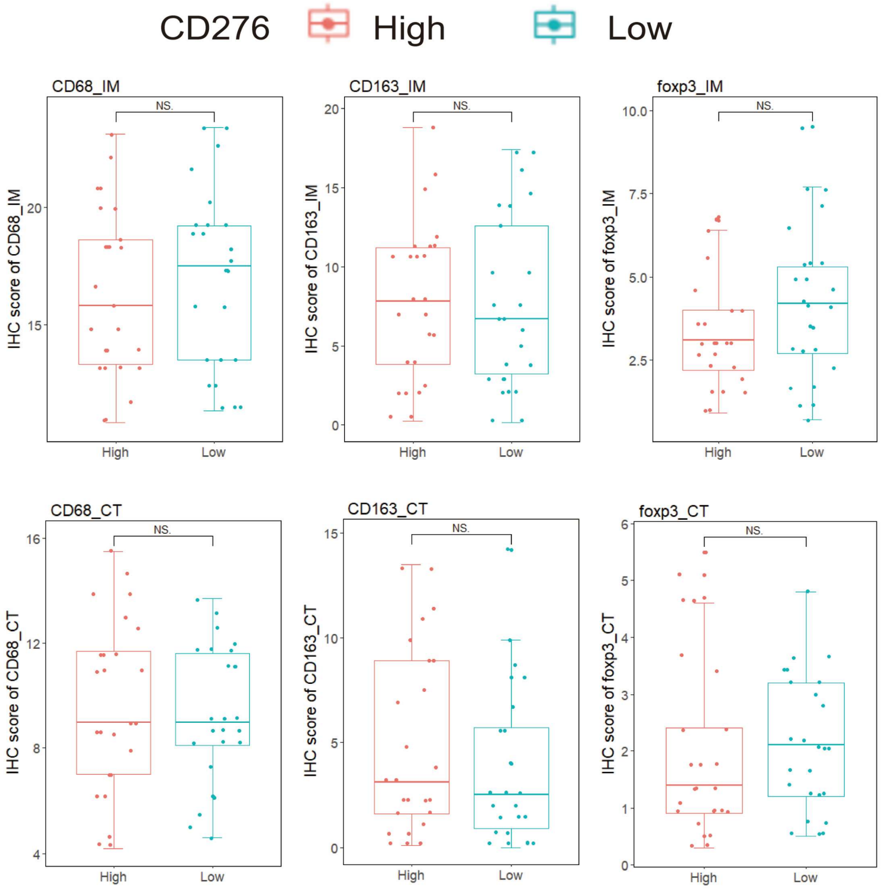

Supplementary Figure 2

A

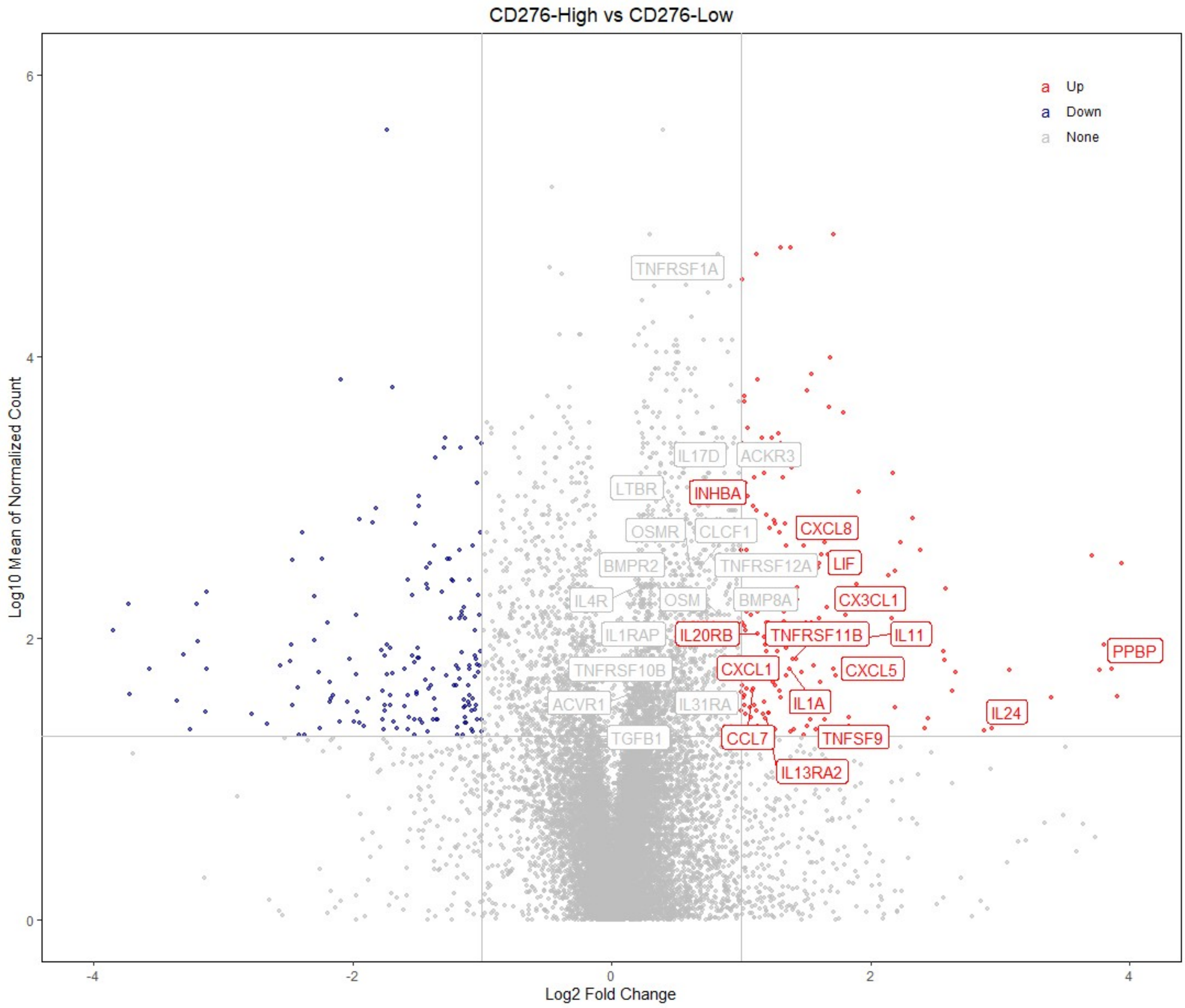

B

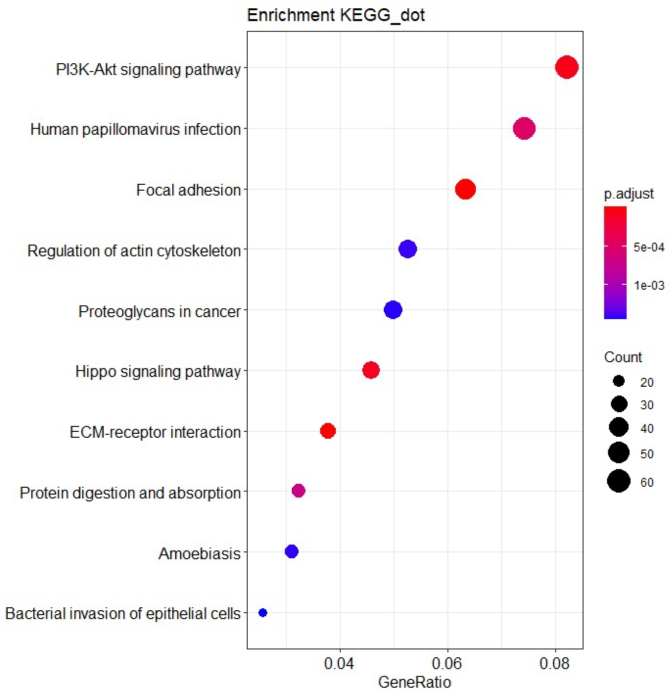

C

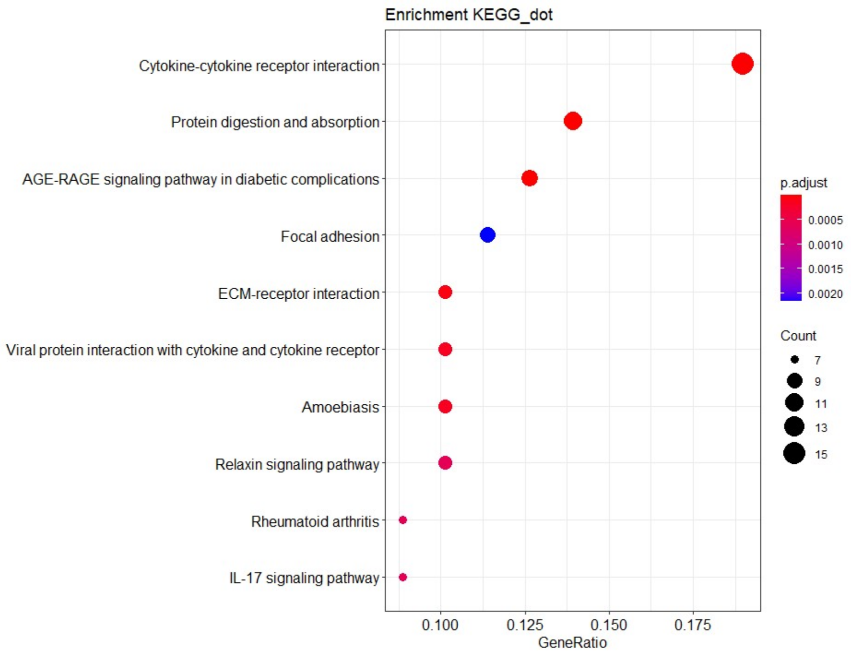

Supplementary Figure 3

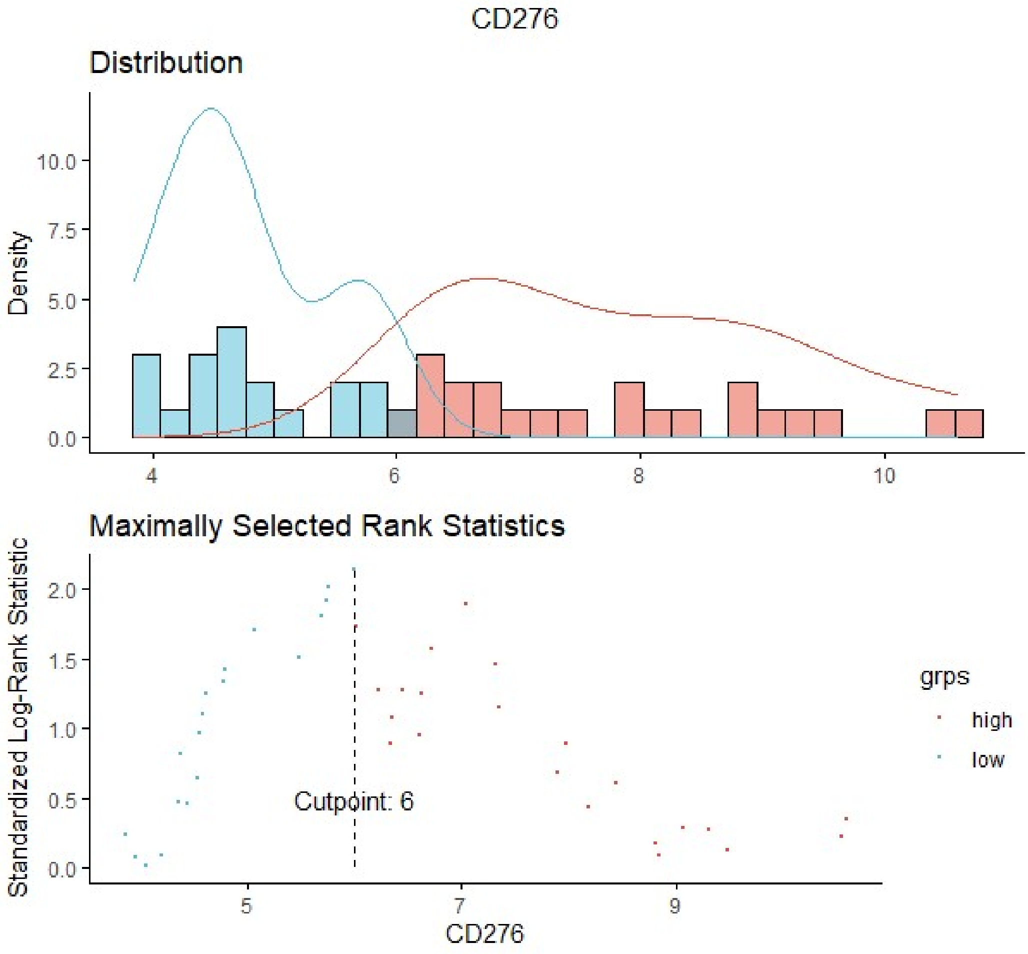

Supplementary Figure 4

A

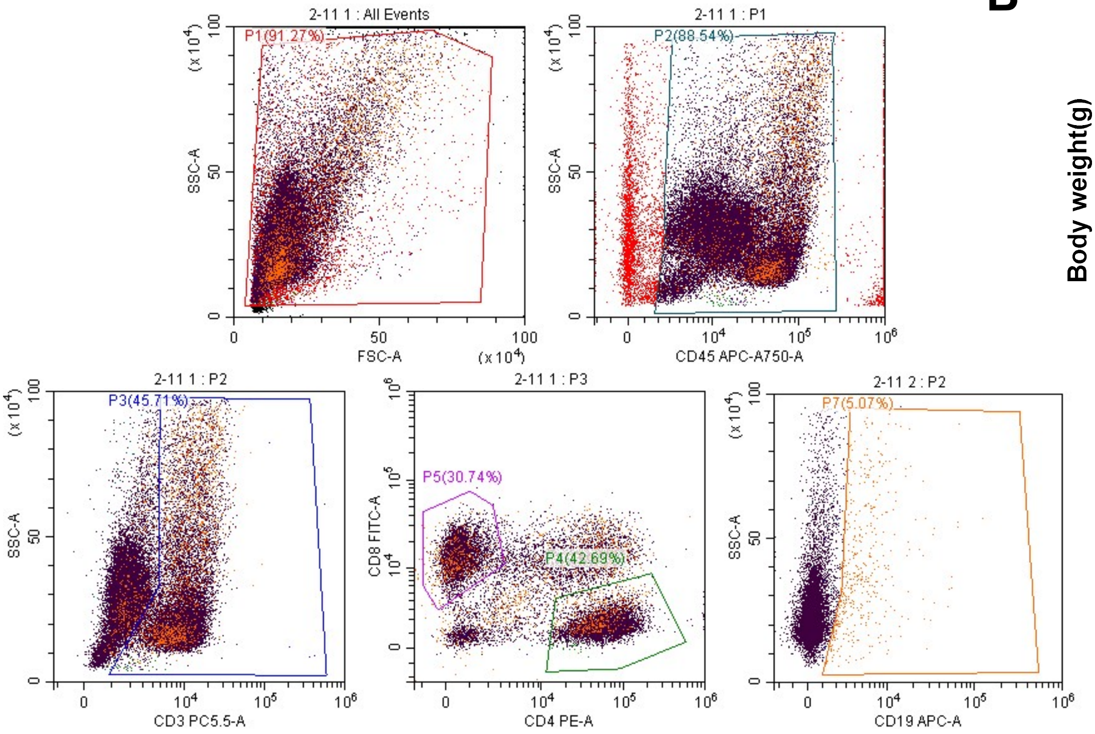

B

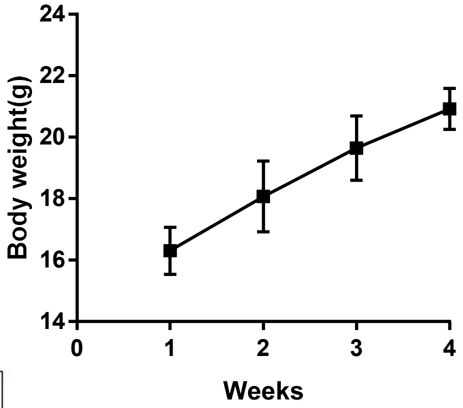

C

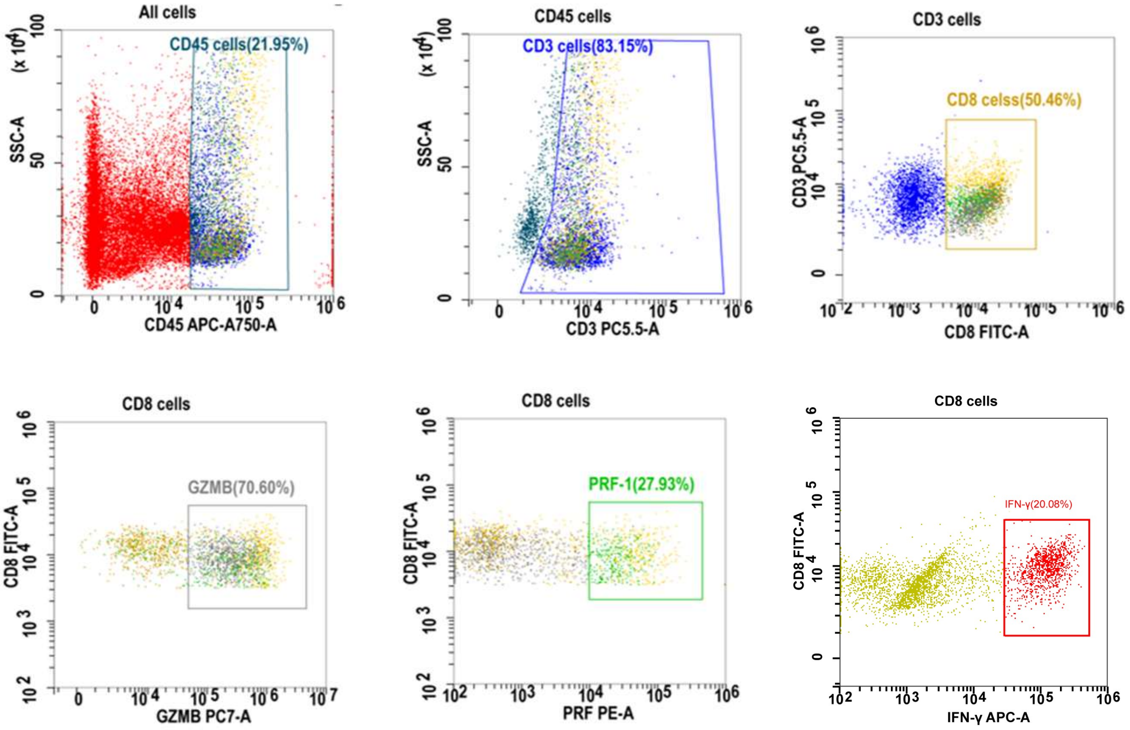

Supplementary Figure 5

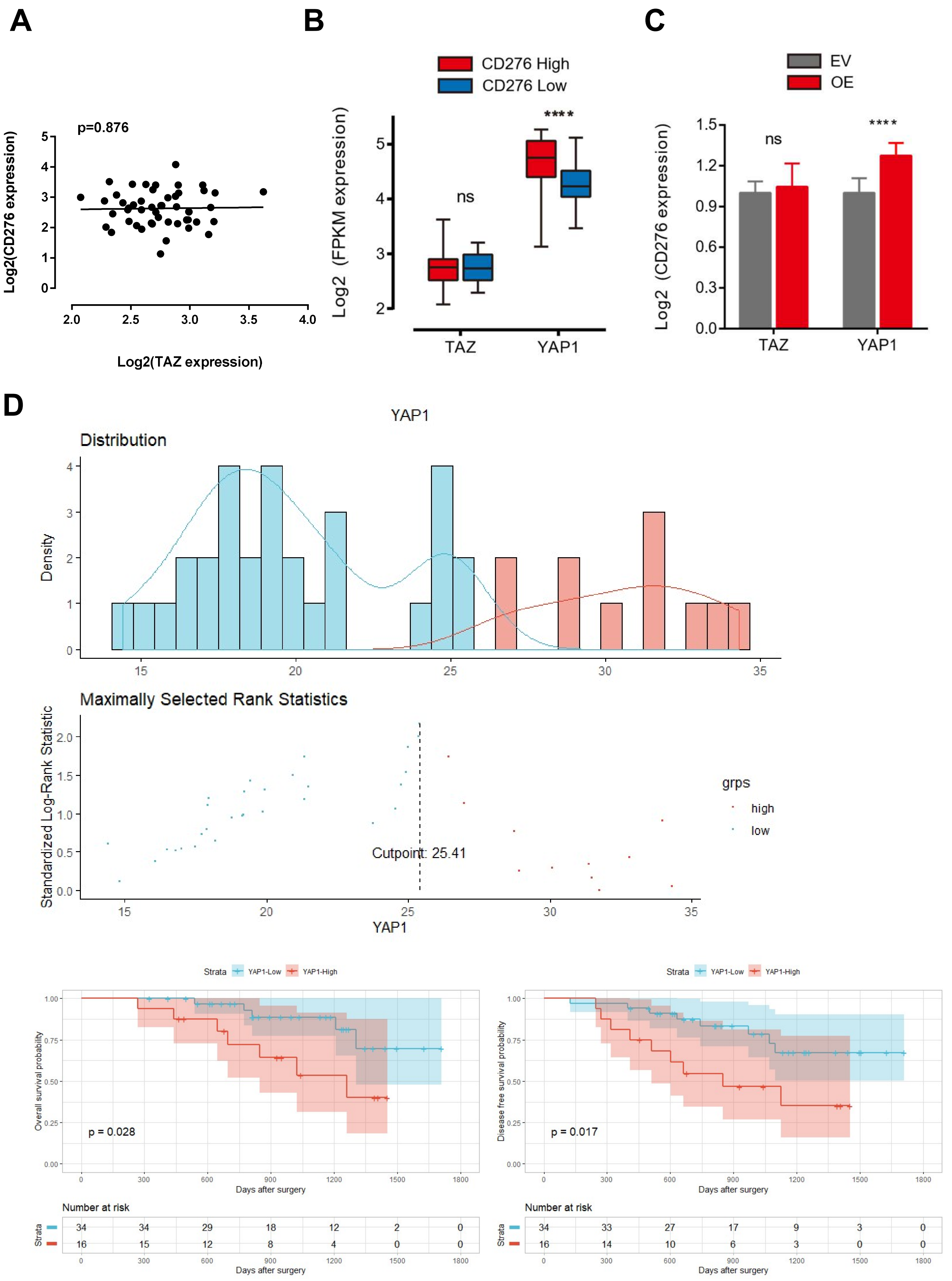

Supplementary Figure 6

A

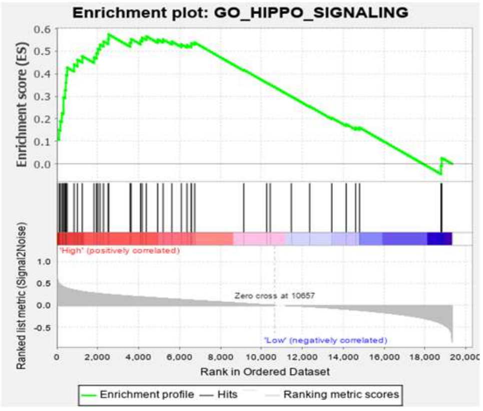

B

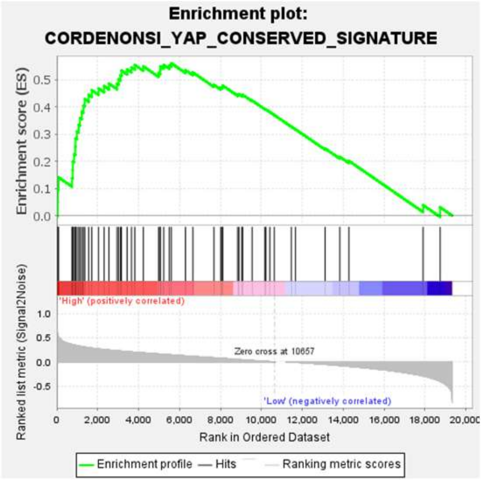

C

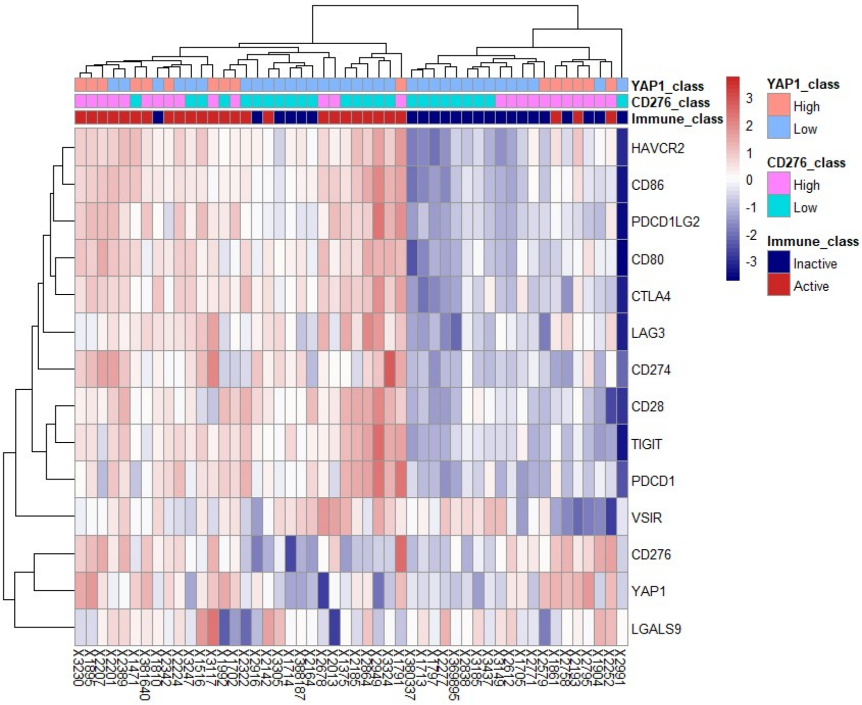

D

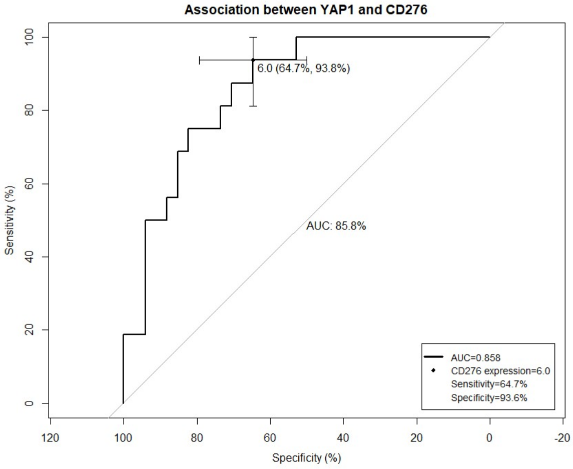

Supplementary Figure 7

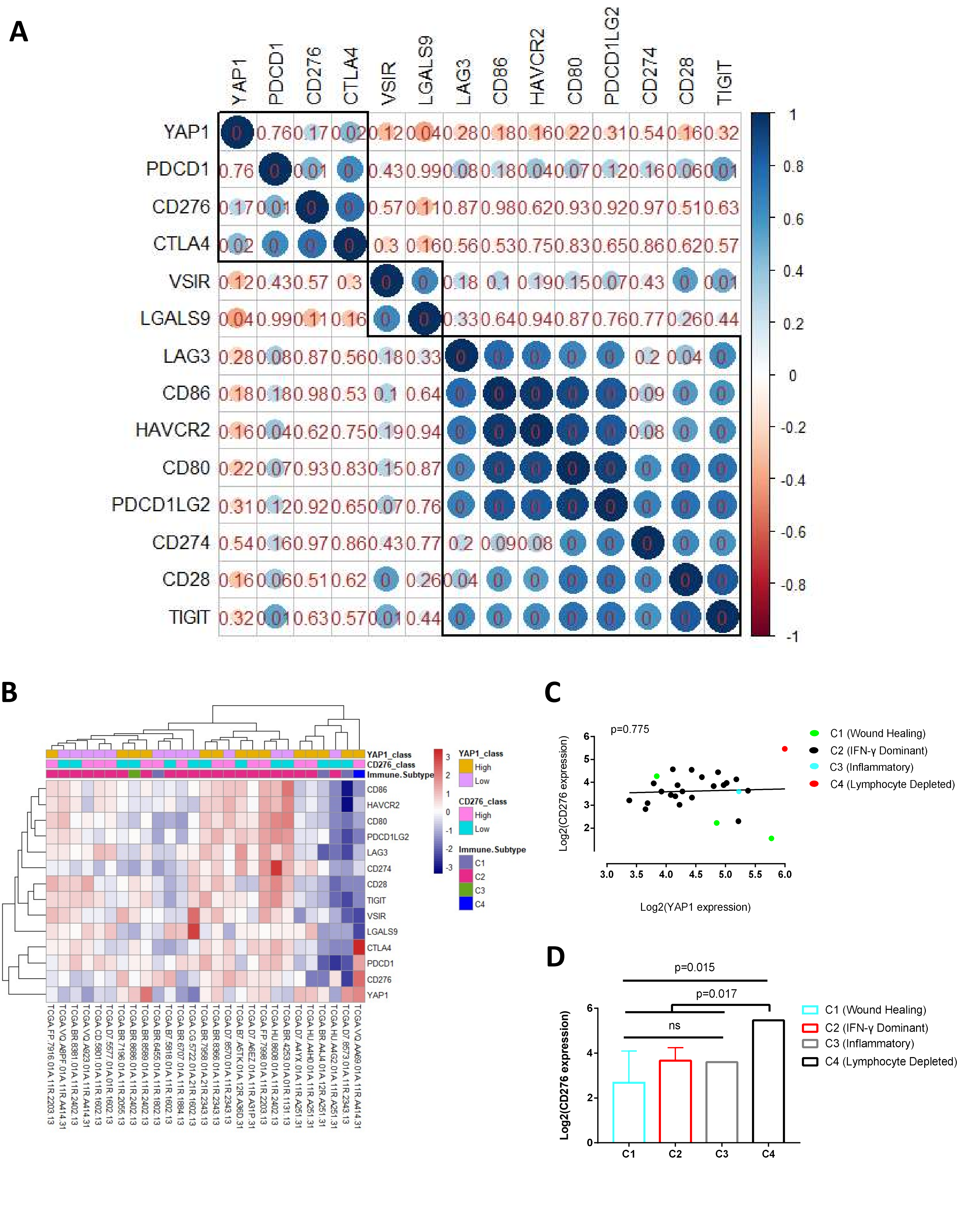

Supplementary Figure 8

Rank	Motif name	PWM	P-value
1	TEAD3	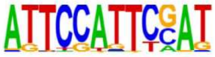	1e-57
2	NFATC1	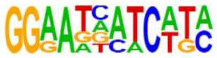	1e-20
3	PRDM1	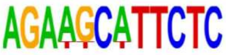	1e-18
4	STAT6	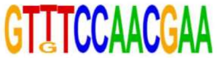	1e-16
5	MSANTD3	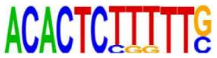	1e-15
6	Isgf3g	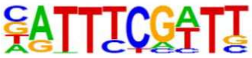	1e-15
7	TEAD4	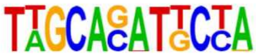	1e-13
8	VDR	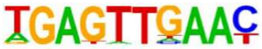	1e-13
9	NKX2.3	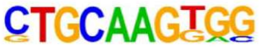	1e-12
10	Ddit3::Cebpa	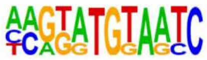	1e-12

Supplementary Figure 9

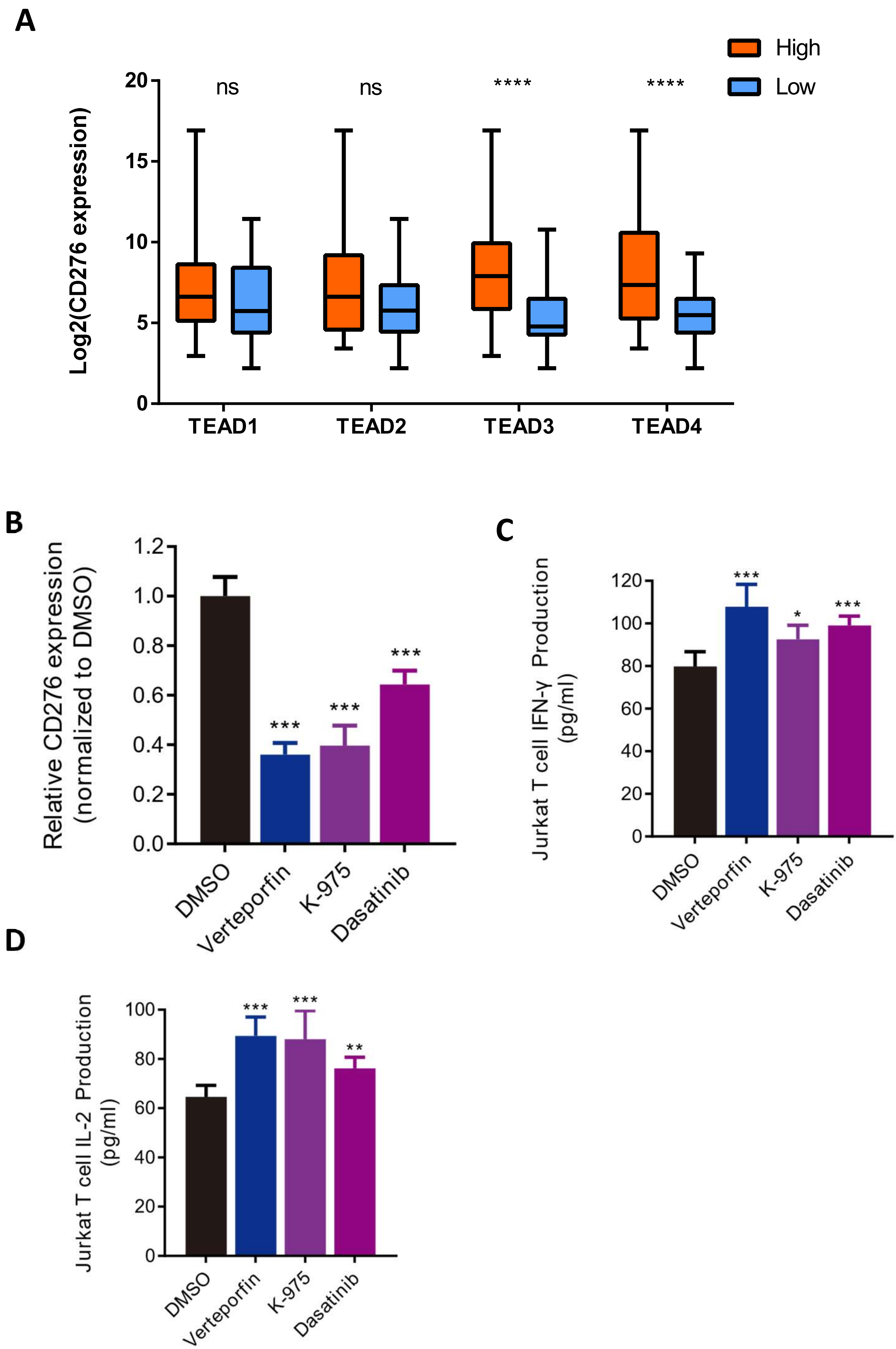

Supplementary Figure 10

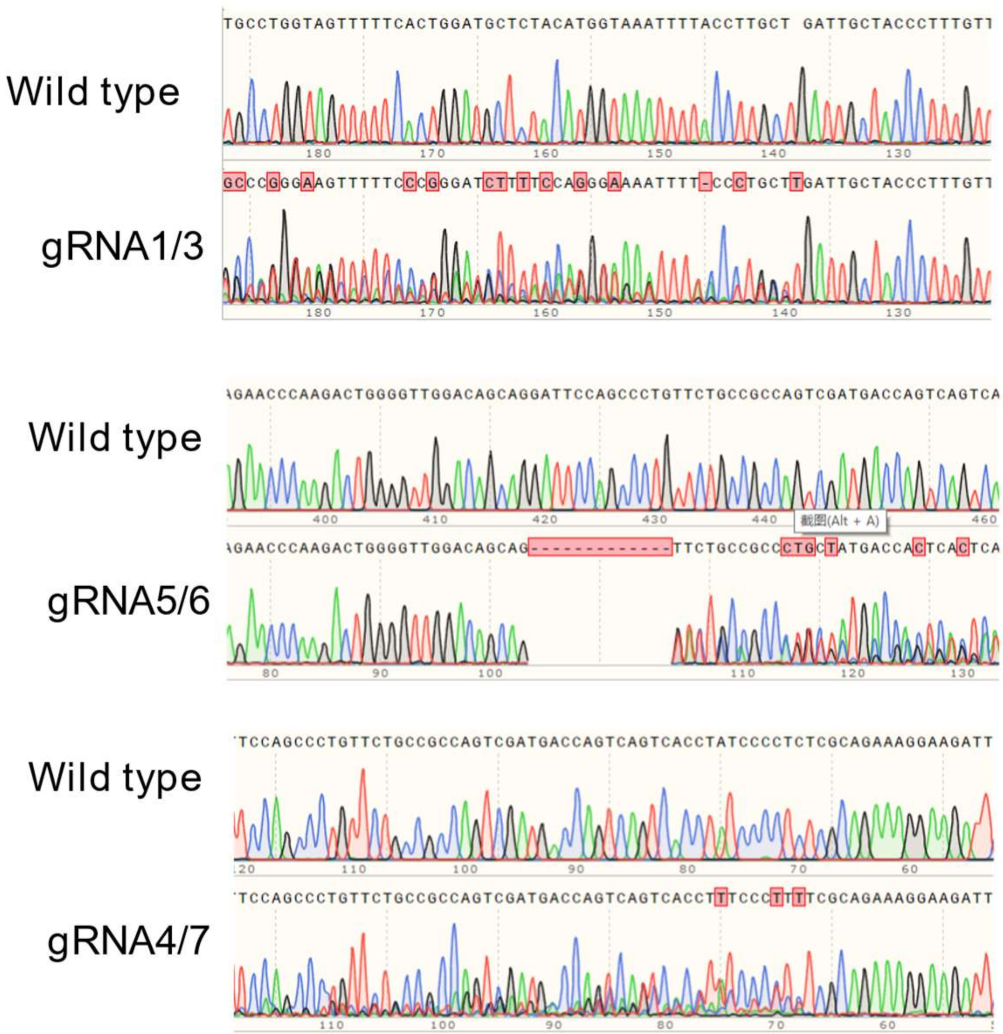

Supplementary Figure 11

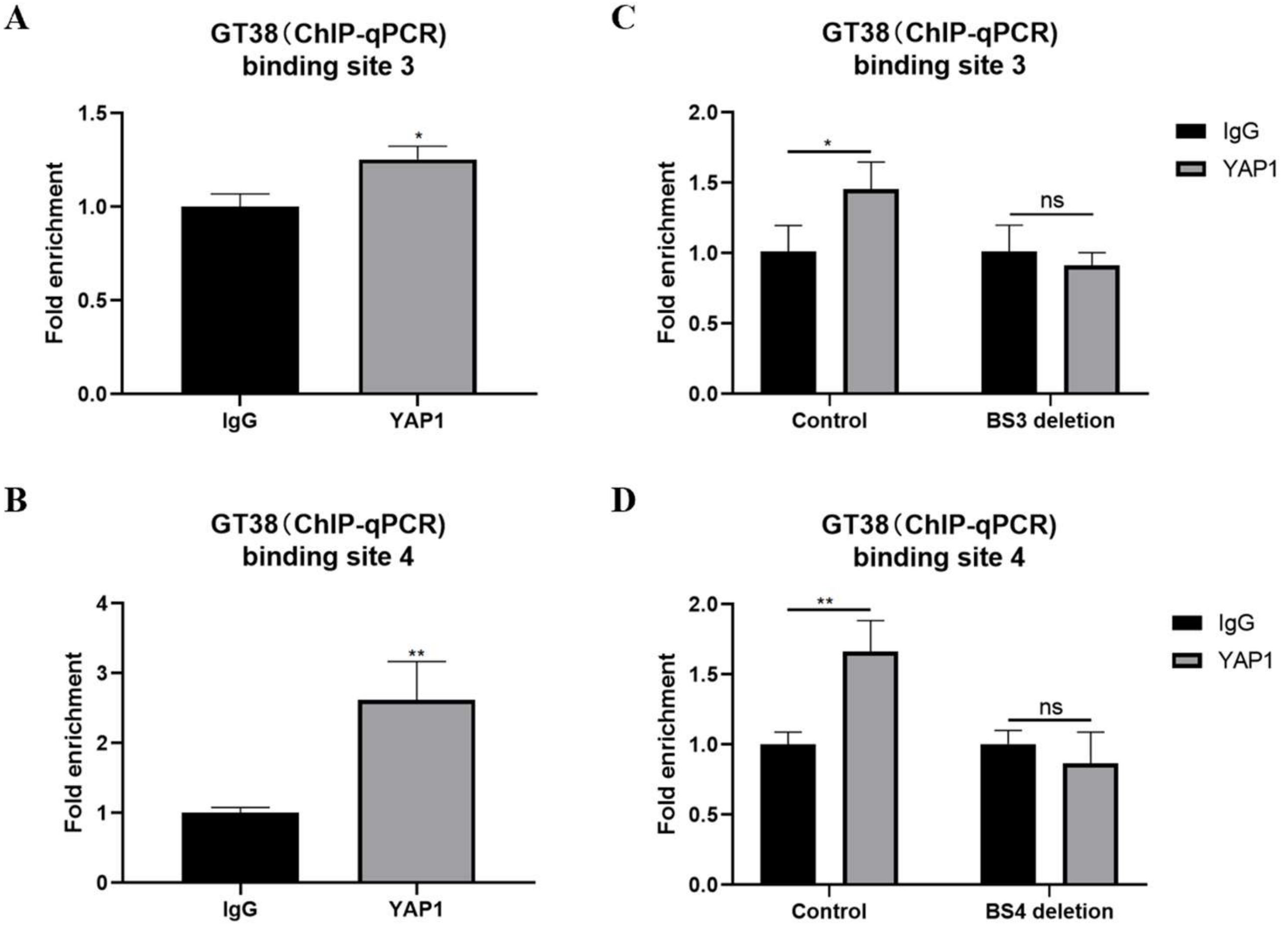

Supplementary Figure 12

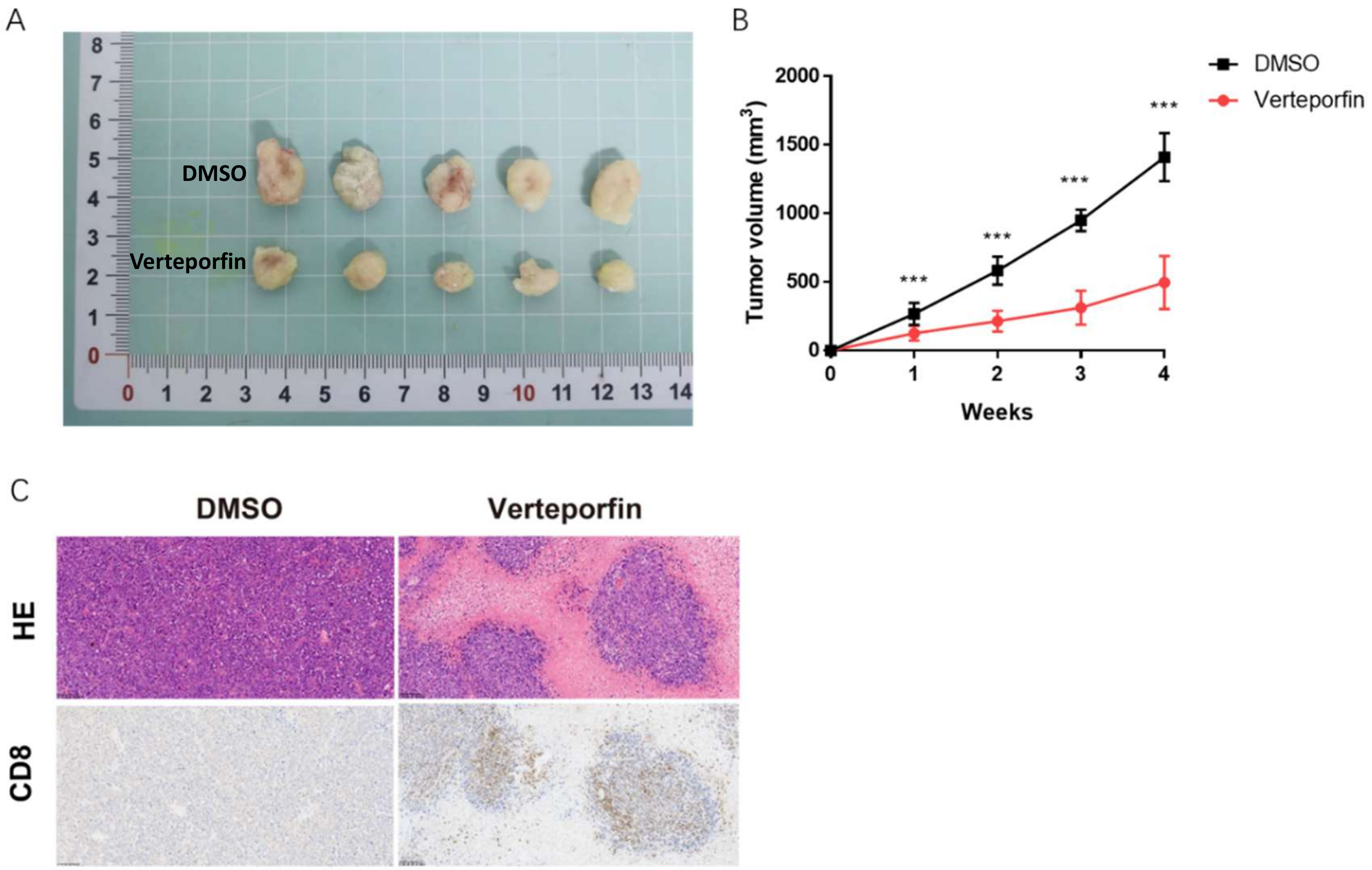

Supplementary Figure 13

Comparison of YAP1 expression between EBV encoded proteins high and low group

EBV encoded transcript	YAP1 expression (Median)		p value
	Transcript_High	Transcript_Low	
A73	24.94	19.18	0.123
BRLF1	-	-	-
EBNA-1	25.19	19.17	0.058
EBNA-2	21.20	21.34	0.802
EBNA-3A	24.75	19.17	0.204
EBNA-3B/EBNA-3C	24.94	21.34	0.800
LMP-1	25.34	21.34	0.720
LMP-2A	24.75	21.34	0.840
RPMS1	24.75	19.23	0.282
Others	25.00	18.20	0.012
