# Supplementary material for: An EBNA1-YAP signaling axis drives immune escape through CD276 in EBV-associated gastric cancer
Source: Cell Death Dis. 2025 Dec 19;17(1):118. doi: 10.1038/s41419-025-08251-2 (PMC12847768; doi:10.1038/s41419-025-08251-2)
Supplement: Supplementary file 2 — Supplementary table 1-2 [file 41419_2025_8251_MOESM2_ESM.docx]

Table S1. Summary of clinical characteristics of patients with EBVaGC

| **Characteristics** | **Count** | **CD276 expression (N=50)** | |  | **P value** |
| --- | --- | --- | --- | --- | --- |
|  |  | **High (N=27)** | **Low (N=23)** |  |  |
| **Sex** |  |  |  |  | 0.054 |
| **Male** | 46 | 23 (85.2%) | 23 (100.0%) |  |  |
| **Female** | 4 | 4 (15.8%) | 0 (0.0%) |  |  |
| **Age** |  |  |  |  | 0.091 |
| **<60** | 17 | 12 (44.4%) | 5 (21.7%) |  |  |
| **≥60** | 33 | 15 (55.6%) | 18 (78.3%) |  |  |
| **Tumor location** |  |  |  |  | 0.421 |
| **Upper** | 14 | 6 (22.2%) | 8 (34.8%) |  |  |
| **Middle** | 26 | 14 (51.9%) | 12 (52.2%) |  |  |
| **Lower** | 10 | 7 (25.9%) | 3 (13.0%) |  |  |
| **Tumor size (cm)** |  |  |  |  | 0.004 |
| **Median** | 4.3 | 5.0 | 3.5 |  |  |
| **Range** | 1.5-13 | 1.5-13 | 1.5-7 |  |  |
| **Lauren type** |  |  |  |  | 0.081 |
| **Intestinal** | 9 | 5 (18.5%) | 8 (34.8%) |  |  |
| **Diffuse** | 24 | 11 (40.7%) | 12 (52.2%) |  |  |
| **Mixed** | 27 | 11 (40.7%) | 3 (13.0%) |  |  |
| **Stage** |  |  |  |  | 0.461 |
| **I+II** | 29 | 9 (33.3%) | 10 (43.5%) |  |  |
| **III+IV** | 31 | 18 (66.7%) | 13 (56.5%) |  |  |
| Data are presented as numbers and rates unless otherwise specified.  The chi-square test or Fisher’s exact test was used for categorical variables, while the Mann–Whitney U test was used for tumor size. | | | | | |

Table S2. Antibodies for western blotting and immunohistochemistry

| **Antibody name** | **Details** |
| --- | --- |
| CD276/B7-H3 | Cell Signaling Technology, CST-14058, 1:1000 |
| YAP | Cell Signaling Technology, CST-14074, 1:1000 |
| p-YAP | Cell Signaling Technology, CST-13008, 1:1000 |
| Flag | Cell Signaling Technology, CST-14793, 1:1000 |
| GAPDH | Cell Signaling Technology, CST-5174, 1:1000 |
| Histone H3 | Cell Signaling Technology, CST-4499S, 1:2000 |
| β-tubulin | Abways, Q13509, 1:20000 |
| p-MST1/2 | Cell Signaling Technology, CST-49332S, 1:1000 |
| MST1/2 | Absin, abs137907, 1:1000 |
| p-LATS1/2 | Absin, abs139992,1:1000 |
| LATS1/2 | Absin, abs137003,1:1000 |
| TEAD4 | Absin, abs954, 1:1000 |
| (IHC primary antibodies) |  |
| CD8 | Cell Signaling Technology, CST-85336, 1:200 |
| CD20 | Cell Signaling Technology, CST-48750, 1:200 |
| CD68 | Cell Signaling Technology, CST-76437, 1:200 |
| CD163 | Cell Signaling Technology, CST-93498, 1:200 |
| Foxp3 | Cell Signaling Technology, CST-12653, 1:200 |
| Ki67 | Cell Signaling Technology, CST- 9449, 1:1000 |
| (Second antibodies) |  |
| Anti-rabbit IgG | Cell Signaling Technology, CST-7074, 1:1000 |
| Anti-mouse IgG | Cell Signaling Technology, CST-7076, 1:1000 |
