# Supplementary material for: An EBNA1-YAP signaling axis drives immune escape through CD276 in EBV-associated gastric cancer
Source: Cell Death Dis. 2025 Dec 19;17(1):118. doi: 10.1038/s41419-025-08251-2 (PMC12847768; doi:10.1038/s41419-025-08251-2)
Supplement: Supplementary file 4 — Supplementary material legends [file 41419_2025_8251_MOESM4_ESM.docx]

**Supplementary Figures**

**Supplementary Figure 1. Effects of CD276 on immune cell components.**

**(A)** Immune cell infiltration scores calculated using the “MCP-Counter” method from RNA-sequencing data of 50 EBV-associated gastric cancer (EBVaGC) samples, highlighting the effects of CD276 on specific immune cell types.

**(B)** High CD276 expression showed no significant correlation with other immune cell types.

**Supplementary Figure 2. Differentially expressed genes and pathways between CD276-High and CD276-Low groups.**

**(A)** Analysis of differentially expressed genes between the CD276-High and CD276-Low groups.

**(B, C)** Pathway enrichment analysis highlighting significantly enriched pathways in the CD276-High group compared to the CD276-Low group.

**Supplementary Figure 3. Determination of the prognostic value of CD276.**

The distribution of CD276 expression values is shown (upper), along with the selection of the cutoff threshold for evaluating the prognostic role of CD276 (lower). The cutoff point was determined based on the value with the maximal standardized Log-Rank statistic.

**Supplementary Figure 4. Establishing immune humanized mice and validating the immune suppressive function of CD276.**

**(A)** Flow cytometric analysis of lymphocyte components in the peripheral blood of immune humanized mice.

**(B)** Weekly body weight measurements to monitor for severe graft-related rejection.

**(C)** Flow cytometric analysis of fresh tissue samples from 15 EBVaGC patients, assessing the immune suppressive function of CD276.

**Supplementary Figure 5. Regulatory and prognostic significance of YAP1 and TAZ expression.**

**(A)** Analysis of RNA sequencing data from 50 EBV-associated gastric cancer (EBVaGC) samples revealed no significant correlation between TAZ expression and CD276 (p = 0.867).

**(B)** YAP1 expression levels were significantly higher in tumors with elevated CD276 expression (p < 0.001).

**(C)** Real-time PCR analysis in the SNU-719 cell line showed that YAP1 overexpression significantly increased CD276 RNA expression (p < 0.001), whereas TAZ overexpression did not result in notable changes.

**(D, upper)** Distribution of YAP1 expression values and selection of the cutoff point for analysis.

**(D, lower)** Prognostic analysis demonstrated that higher YAP1 expression was associated with poorer overall survival (OS) and disease-free survival (DFS) (n = 50, p = 0.028 and p = 0.017, respectively).

**Supplementary Figure 6. Significance of YAP1 expression.**

**(A, B)** Enrichment analysis revealed significant enrichment of the "Hippo signaling" and "YAP conserved signature" pathways in the YAP1-High group.

**(C)** Heatmap showing unsupervised clustering of immune checkpoint molecules and YAP1 expression.

**(D)** Predictive value of CD276 for YAP1 expression, with an AUC of 0.858, sensitivity of 64.7%, and specificity of 93.6%.

**Supplementary Figure 7. Validation of the immune regulatory function of CD276 in TCGA datasets.**

**(A, B)** CD276 is regulated independently of other immune checkpoint molecules.

**(C, D)** Tumors of the lymphocyte-depleted subtype exhibited the highest levels of CD276 and YAP1 expression.

**Supplementary Figure 8. Motif analysis of ChIP-seq peaks in YAP-overexpressing GT38 cells.**

ChIP-seq analysis using an anti-Flag-YAP antibody in GT38 cells identified the DNA-binding motifs of the transcription factors TEAD3 and TEAD4 as being significantly enriched at YAP1-occupied sites.

**Supplementary Figure 9. Correlation between CD276 expression and TEAD family transcription factors.**

**(A)** Higher expression of TEAD3 or TEAD4 was associated with significantly elevated CD276 expression levels (p < 0.001).

**(B)** CD276 expression after treated with verteporfin, K-975 and dasatinib after 24 hours, respectively.

**(C, D)** In co-culture system, inhibitors treated cells increased IFN-γ and IL-2 secretion of activated Jurkat T cells.

**Supplementary Figure 10. Sanger sequencing validation of CRISPR cutting efficiency.**

Sanger sequencing was used to confirm the efficiency of CRISPR-mediated genomic editing.

**Supplementary Figure 11. YAP binding to TEAD4 sites at the CD276 promoter.**

**(A, B)** ChIP-qPCR analysis in GT38 cells demonstrated YAP binding at regulatory elements BS3 and BS4, where TEAD4 also binds.

**(C, D)** CRISPR/Cas9-mediated deletion of BS3 or BS4 reduced YAP1 enrichment at these sites.

**Supplementary Figure 12. Efficacy of YAP-TEAD blockade in vivo.**

**(A, B)** Verteporfin, a YAP-TEAD inhibitor, significantly suppressed tumor growth in humanized xenograft mice.

**(C)** YAP-TEAD blockade increased CD8+ T cell infiltration within the tumor microenvironment.

**Supplementary Figure 13. Correlation between EBV-encoded protein expression and YAP.**

EBNA1 showed the strongest statistical influence on YAP expression, albeit with marginal significance (p = 0.058).

**Supplementary Table 1. Summary of clinical characteristics of patients with EBVaGC.**

**Supplementary Table 2. Antibodies for western blotting and immunohistochemistry.**

**Supplementary Table 3: Oligonucleotide sequences**
