# Supplementary material for: An EBNA1-YAP signaling axis drives immune escape through CD276 in EBV-associated gastric cancer
Source: Cell Death Dis. 2025 Dec 19;17(1):118. doi: 10.1038/s41419-025-08251-2 (PMC12847768; doi:10.1038/s41419-025-08251-2)
Supplement: Supplementary file 5 — original blots image [file 41419_2025_8251_MOESM5_ESM.pptx]

## Slide 1
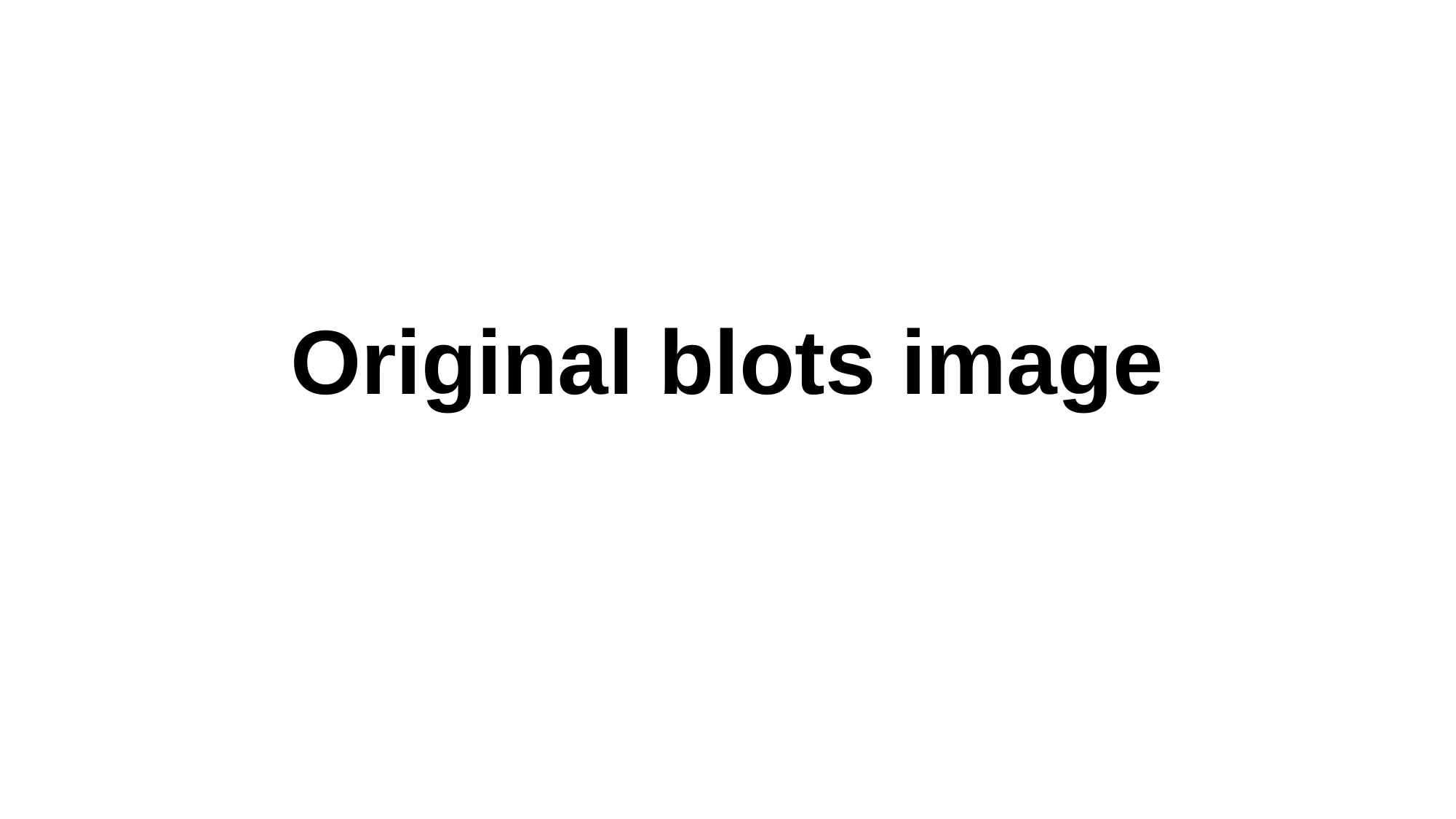

# Original blots image

## Slide 2
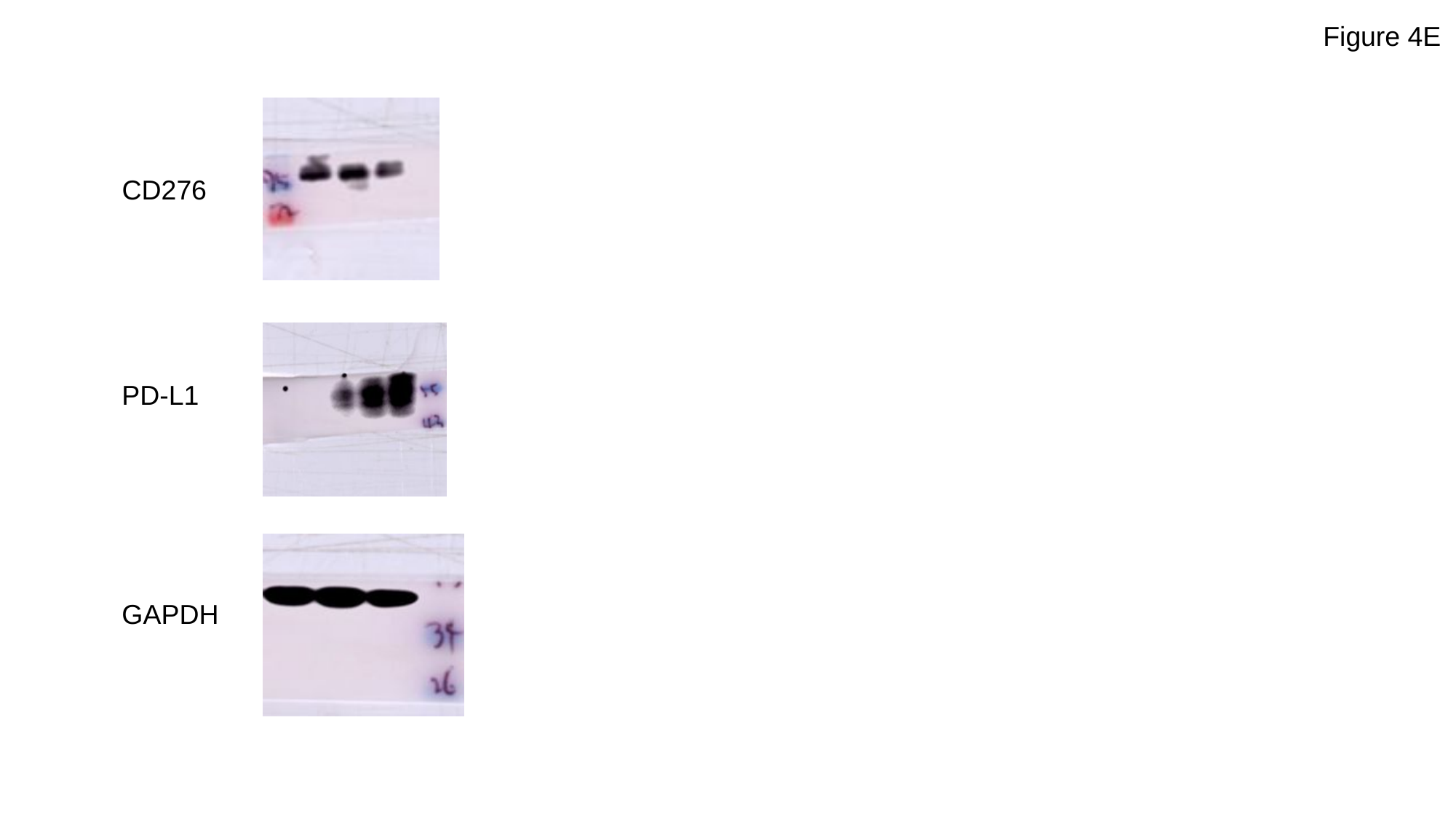

Figure 4E
CD276
PD-L1
GAPDH

## Slide 3
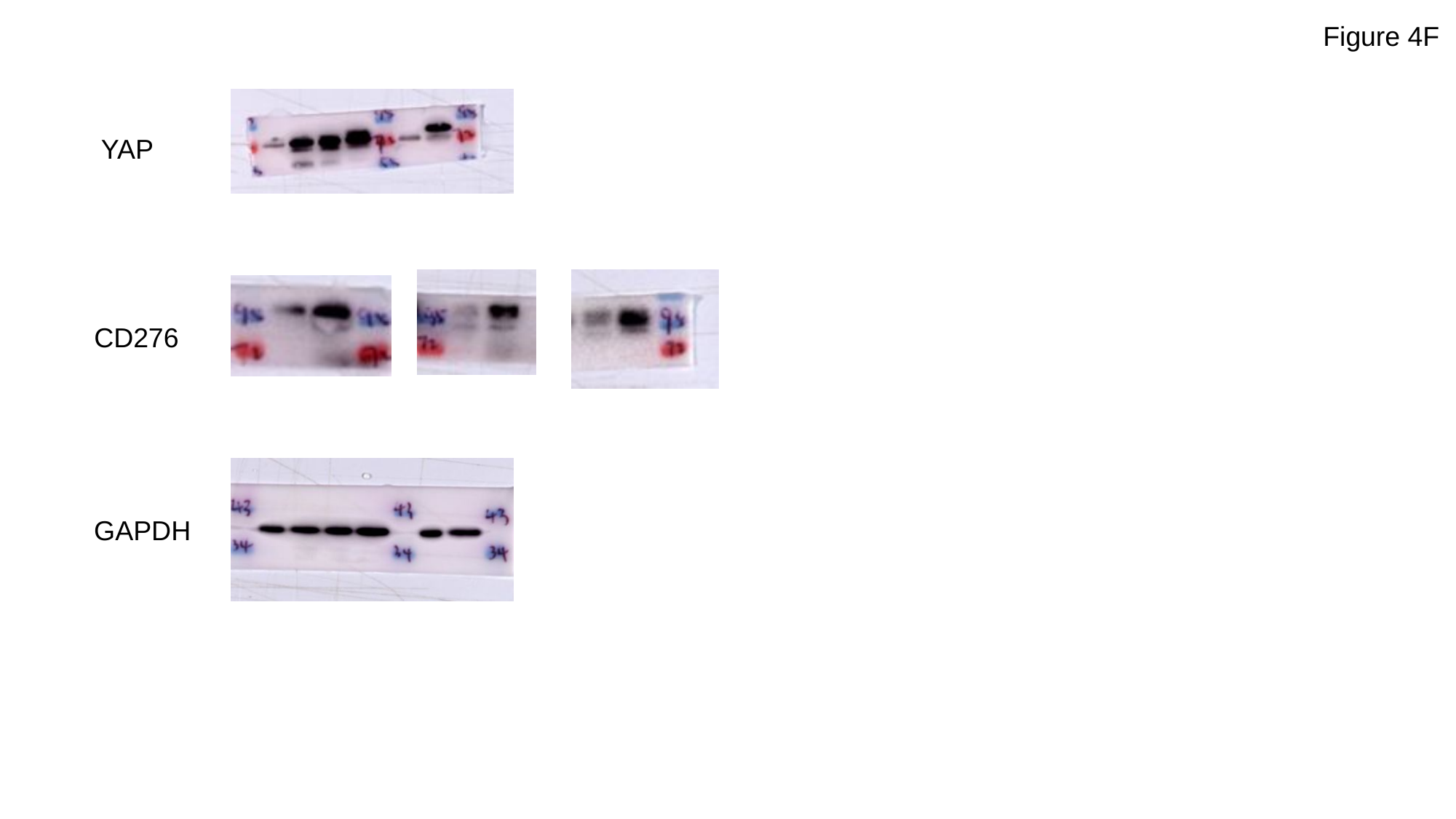

Figure 4F
YAP
CD276
GAPDH

## Slide 4
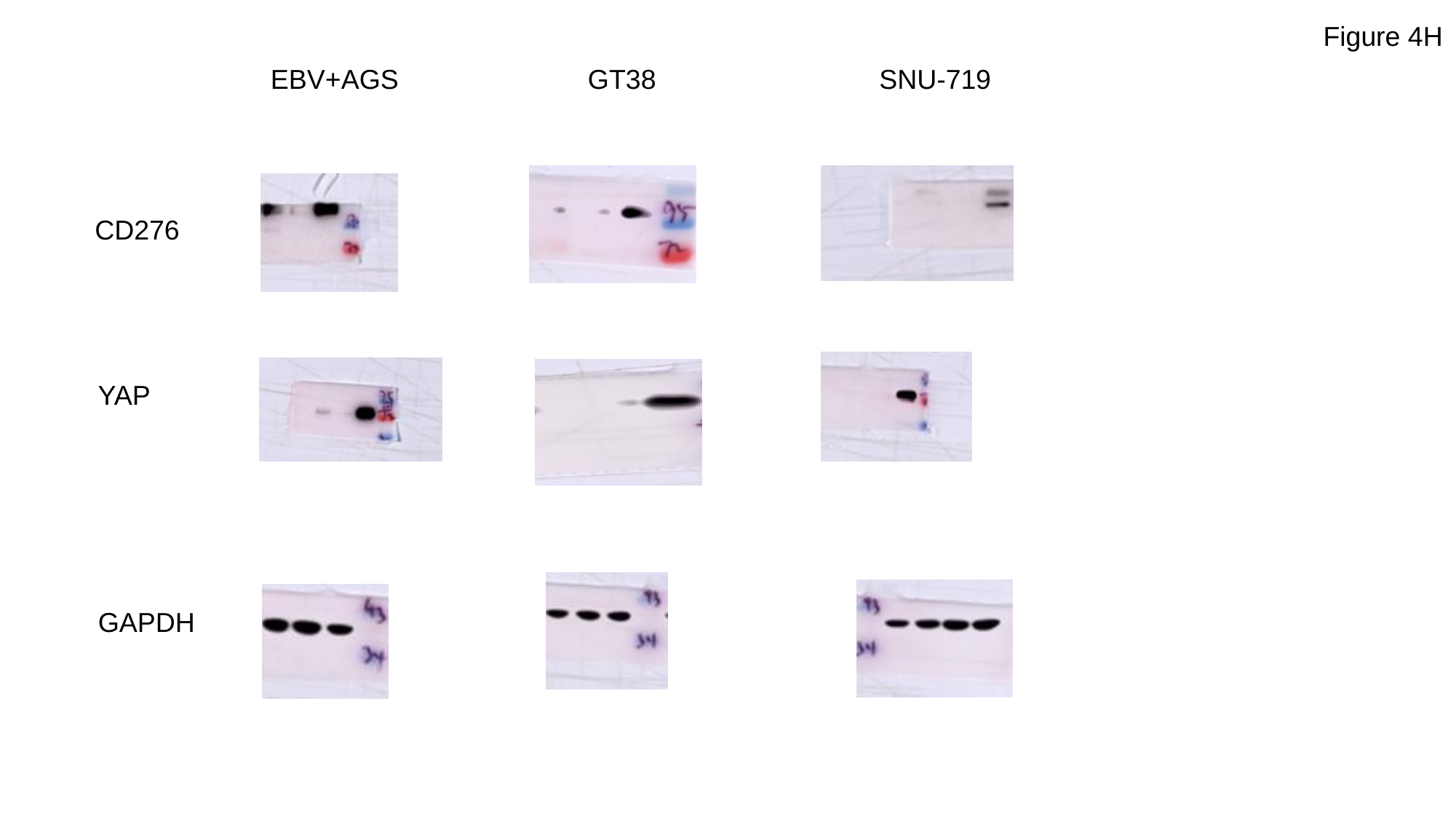

Figure 4H
EBV+AGS
GT38
SNU-719
CD276
YAP
GAPDH

## Slide 5
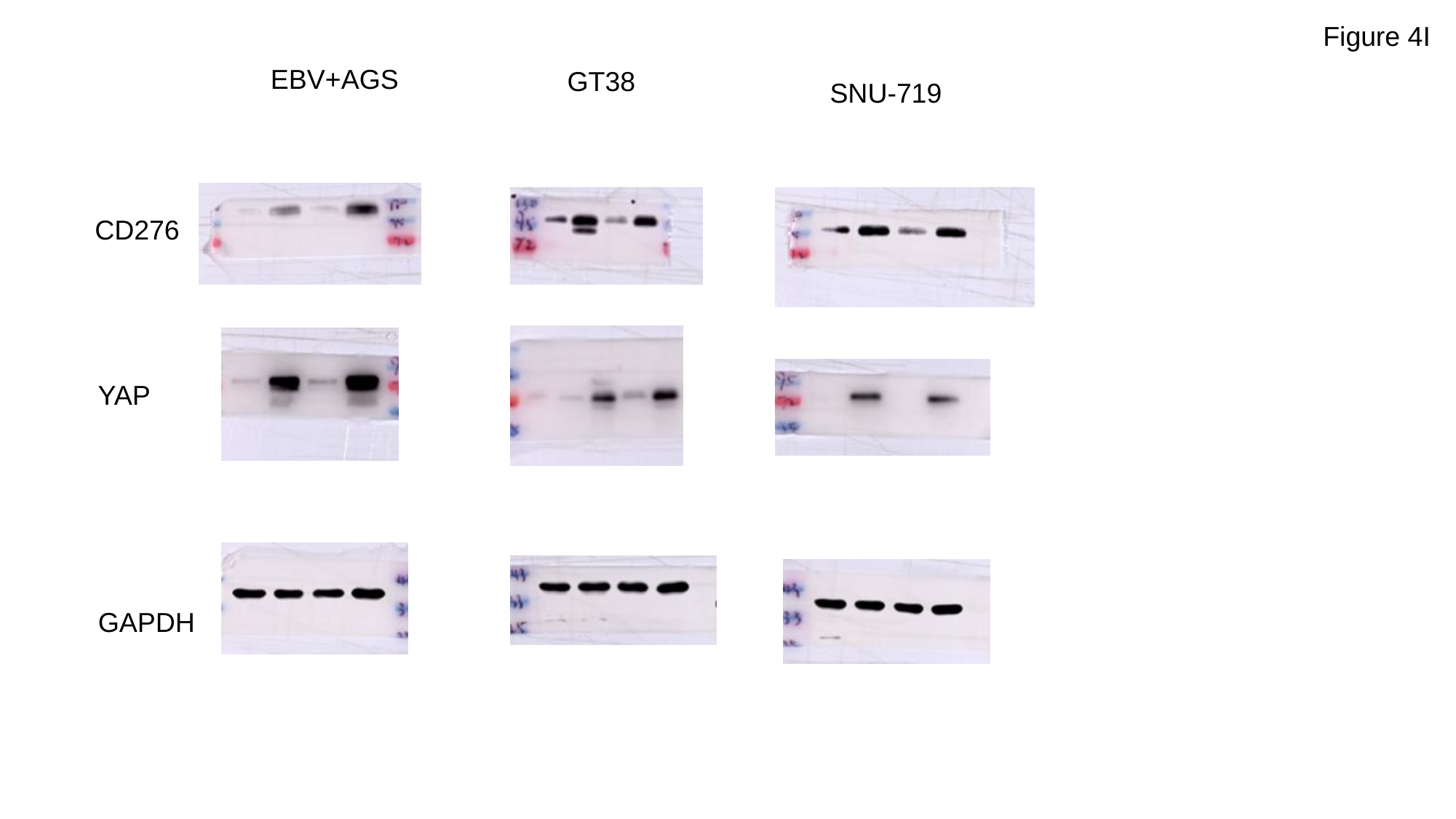

Figure 4I
EBV+AGS
GT38
SNU-719
CD276
YAP
GAPDH

## Slide 6
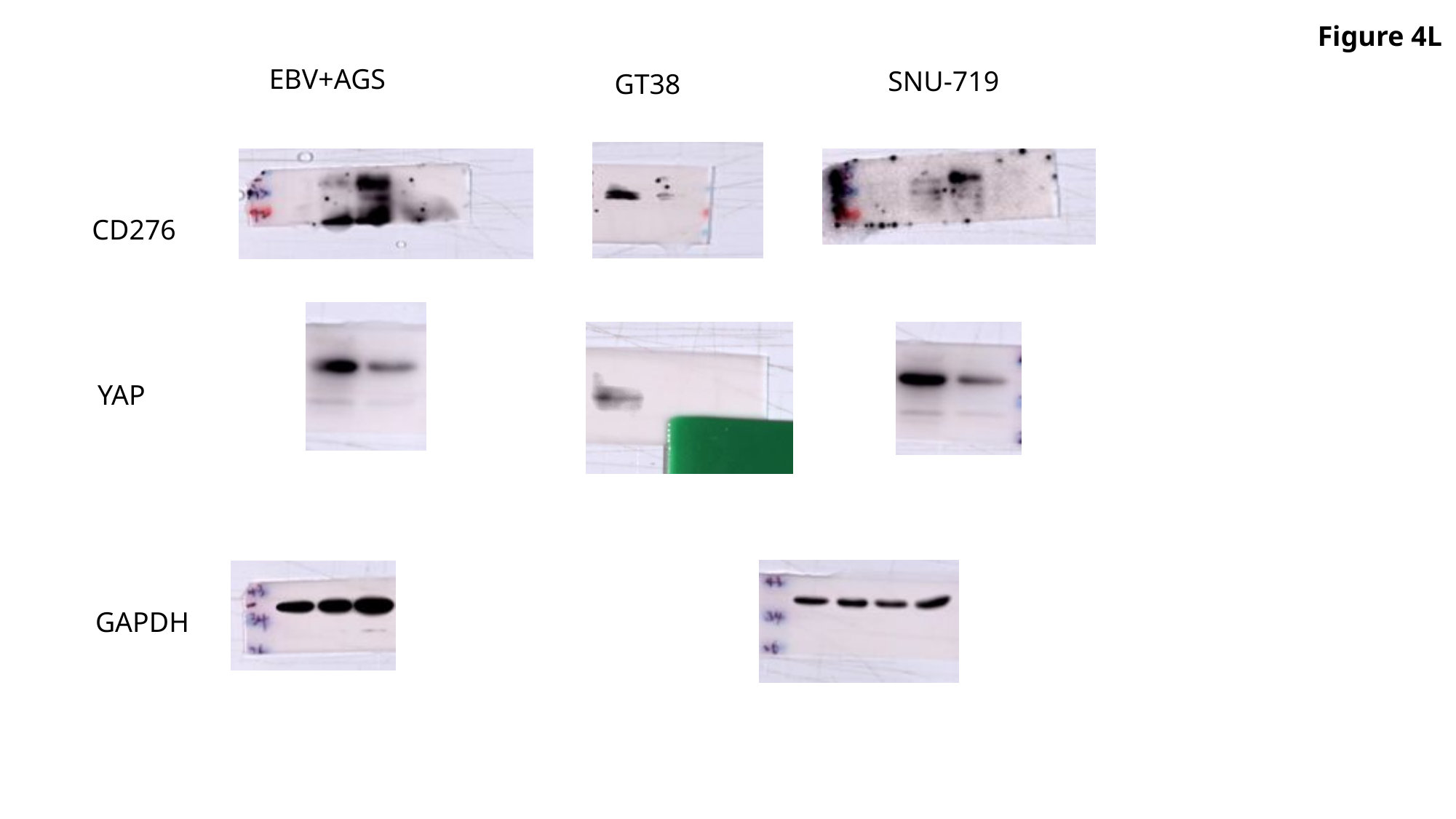

Figure 4L
EBV+AGS
SNU-719
GT38
CD276
YAP
GAPDH

## Slide 7
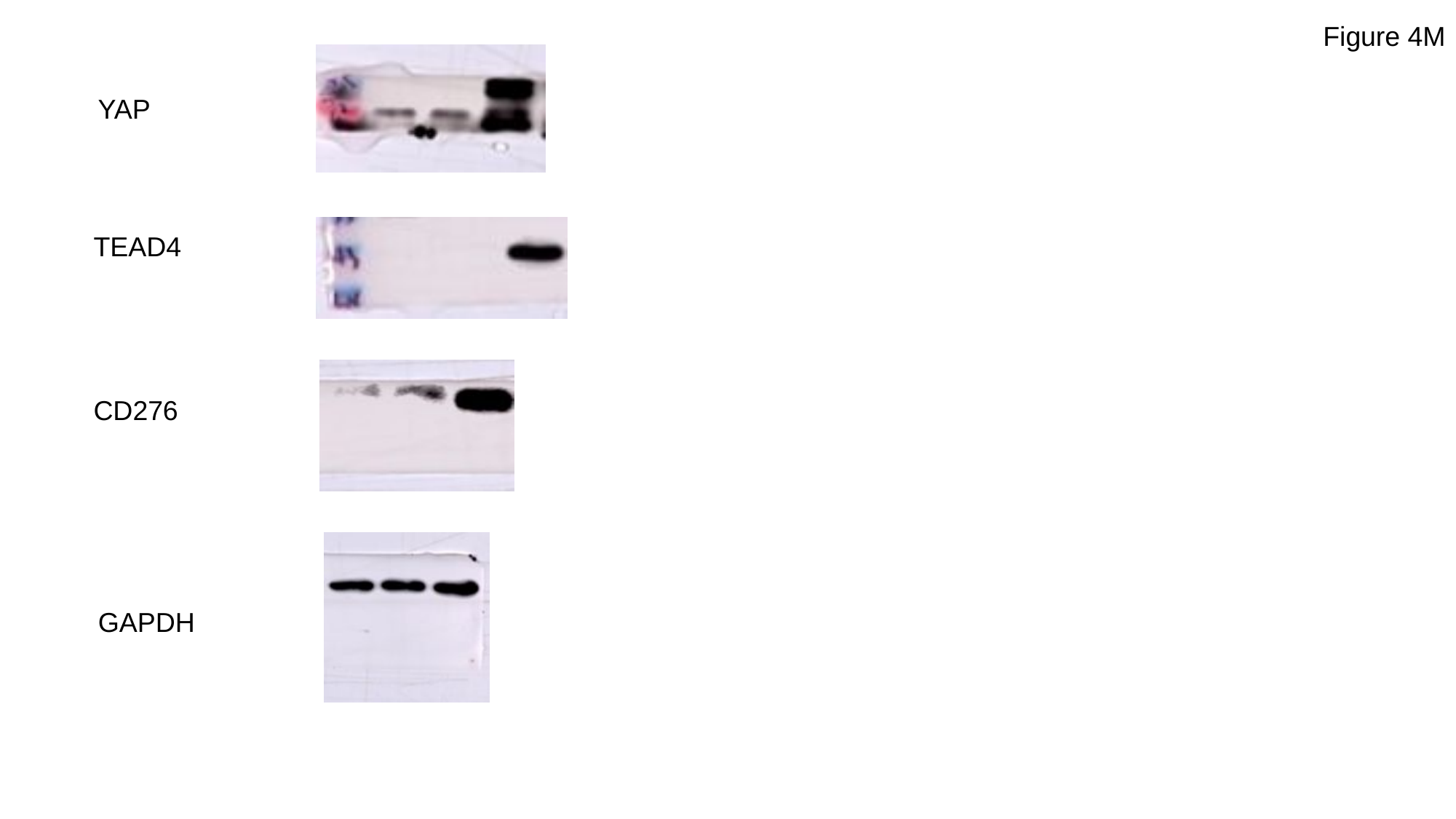

Figure 4M
YAP
TEAD4
CD276
GAPDH

## Slide 8
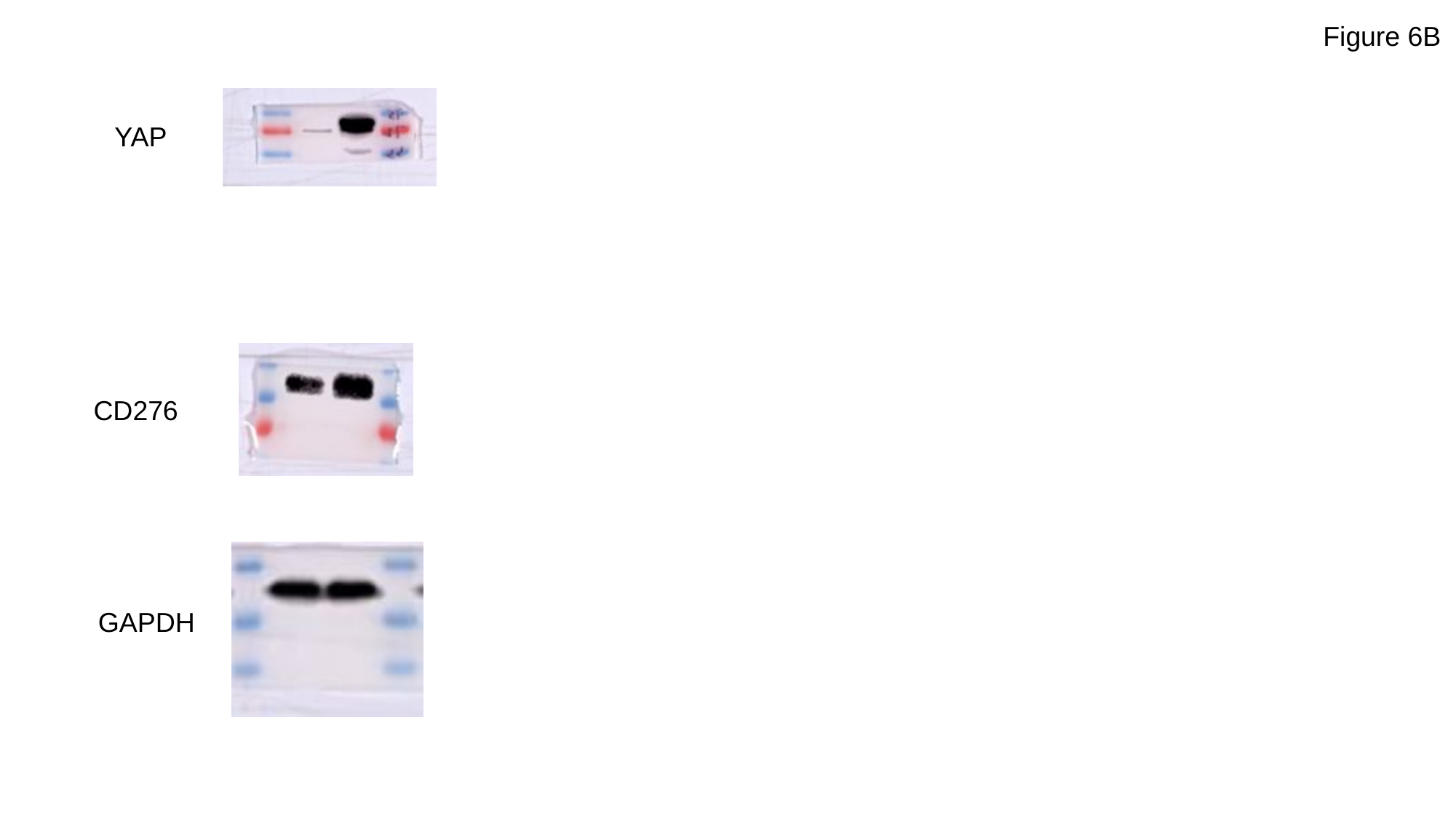

Figure 6B
YAP
CD276
GAPDH

## Slide 9
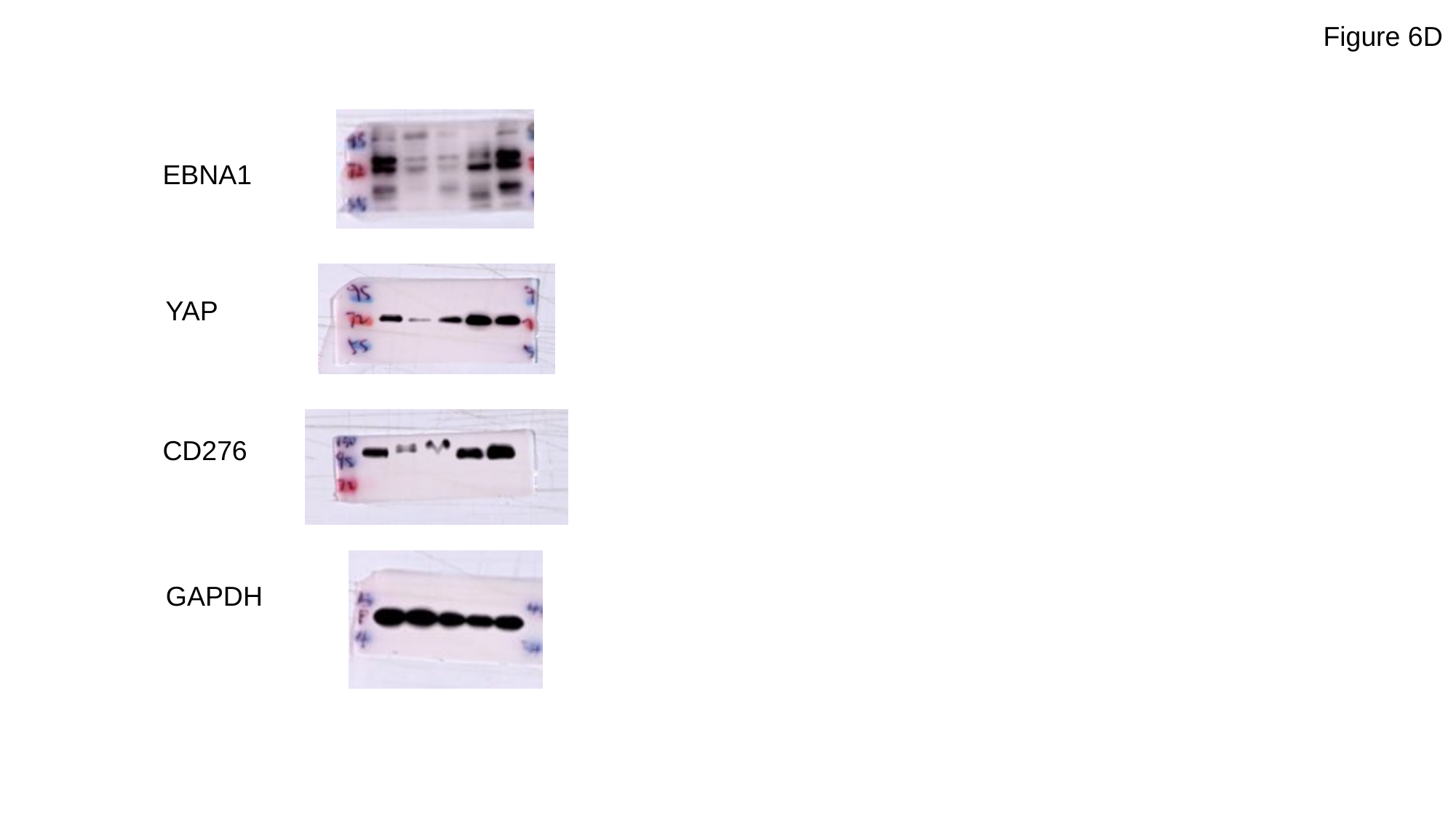

Figure 6D
EBNA1
YAP
CD276
GAPDH

## Slide 10
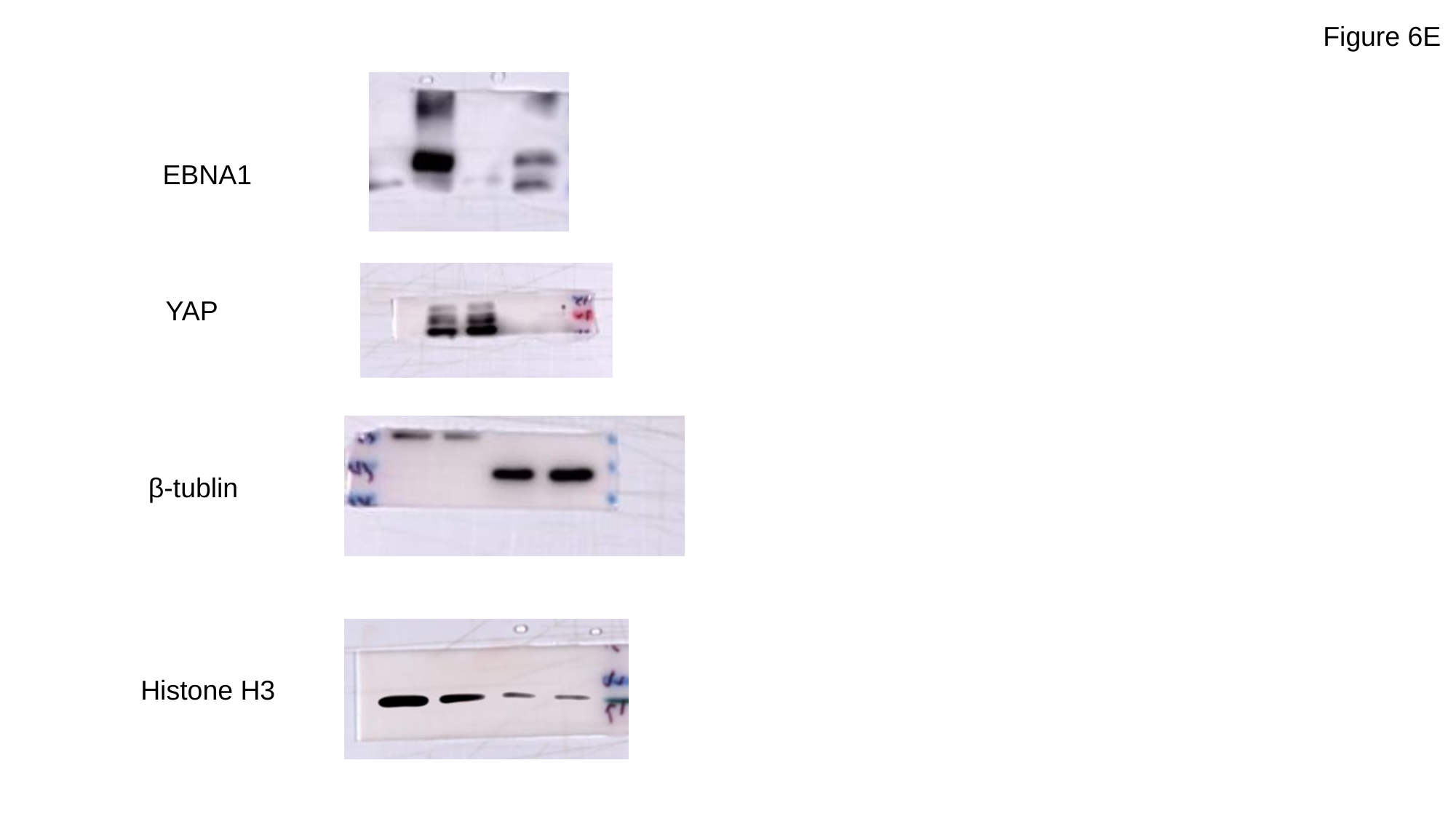

Figure 6E
EBNA1
YAP
β-tublin
Histone H3

## Slide 11
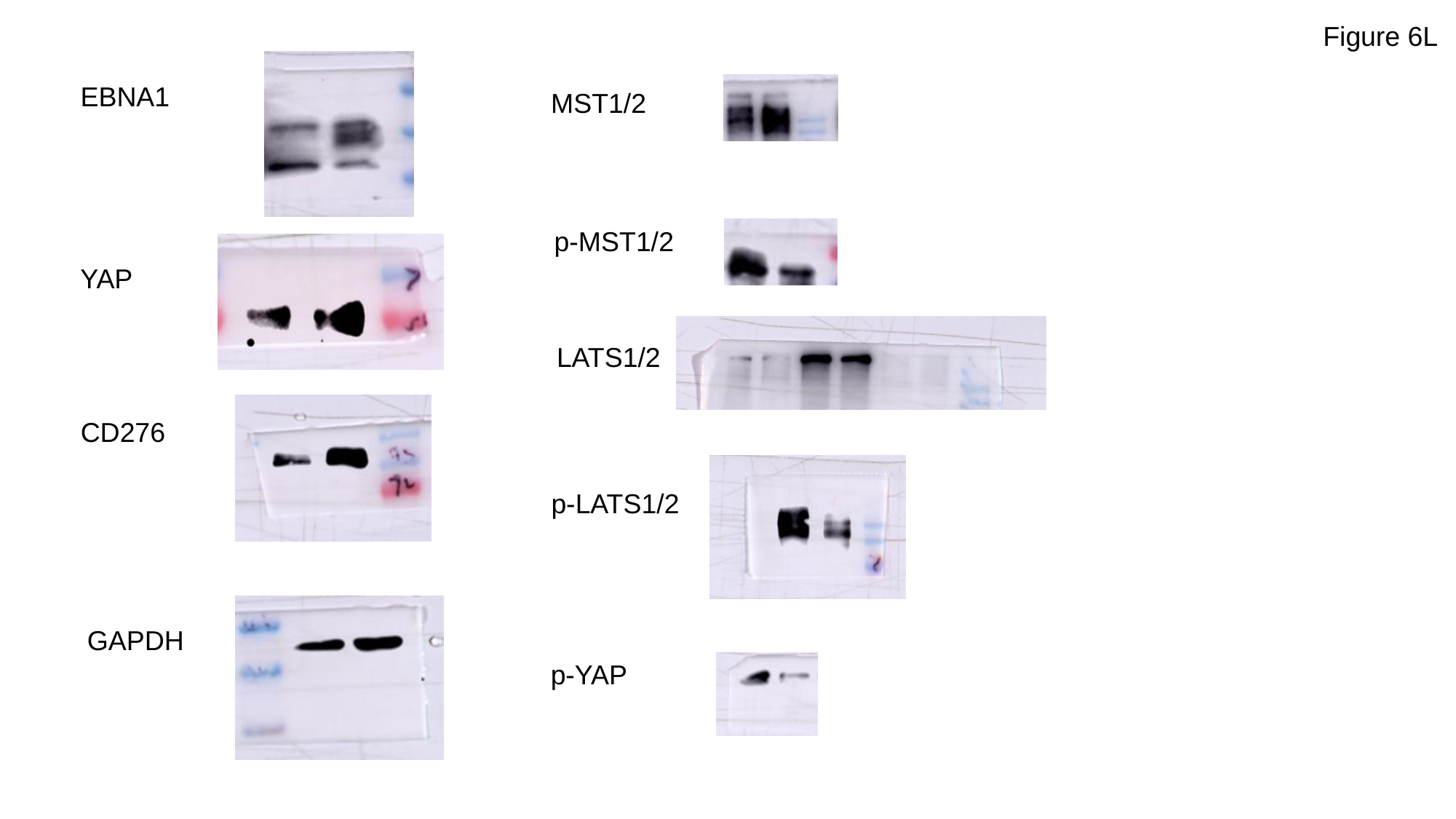

Figure 6L
EBNA1
MST1/2
p-MST1/2
YAP
LATS1/2
CD276
p-LATS1/2
GAPDH
p-YAP

## Slide 12
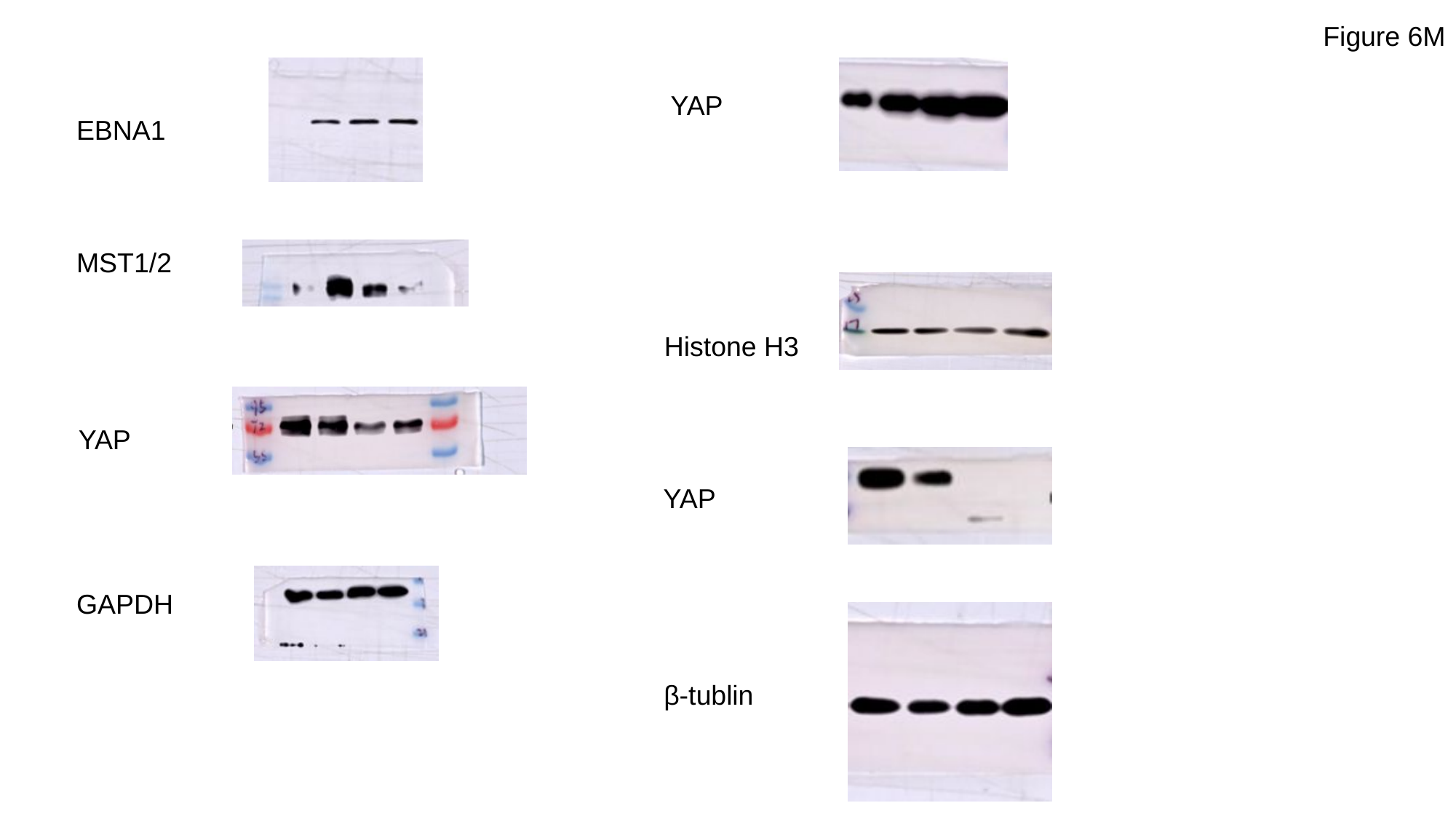

Figure 6M
YAP
EBNA1
MST1/2
Histone H3
YAP
YAP
GAPDH
β-tublin
